# Genetic Encoding of a Trifunctional Photo‐Cross‐Linker with a Cleavable Alkyl Ester Moiety

**DOI:** 10.1002/cbic.202500827

**Published:** 2026-01-30

**Authors:** Masahiro Takayama, Tomoya Tsubota, Takao Yamaguchi, Kosuke Chiba, Takumi Yoshida, Yoshiyuki Hari, Yu‐Shi Tian, Daisuke Takaya, Asuka Mori, Tomohito Tsukamoto, Kenji Ishimoto, Yukio Ago, Yoshiaki Okada, Kensaku Sakamoto, Takefumi Doi, Kaori Fukuzawa, Satoshi Obika, Shinsaku Nakagawa, Nobumasa Hino

**Affiliations:** ^1^ Graduate School of Pharmaceutical Sciences The University of Osaka Suita Japan; ^2^ Shionogi Pharmaceutical Research Center Shionogi & Co. Ltd. Toyonaka Japan; ^3^ Faculty of Pharmaceutical Sciences Tokushima Bunri University Tokushima Japan; ^4^ Department of Cellular and Molecular Pharmacology Graduate School of Biomedical and Health Sciences Hiroshima University Hiroshima Japan; ^5^ Center for Infectious Disease Education and Research The University of Osaka Suita Japan; ^6^ Laboratory for Nonnatural Amino Acid Technology RIKEN Center for Biosystems Dynamics Research RIKEN Yokohama Japan; ^7^ Department of Drug Target Protein Research Shinshu University School of Medicine Matsumoto Japan

**Keywords:** genetic code expansion, noncanonical amino acid, photo‐cross‐linking, protein modifications, protein‐protein interactions

## Abstract

Genetically encoded photo‐cross‐linkable amino acids (PAAs) are powerful tools for analyzing direct protein–protein interactions (PPIs) in mammalian cells. Cleavable PAAs are particularly useful, enabling covalent capture and subsequent release of interacting partners, which facilitates the characterization of interaction interfaces using mass spectrometry. However, the limited options for cleavable linker structures have restricted the design of PAAs. In this study, we genetically encoded a novel trifunctional PAA, DiZAAsu, which contains three distinct chemical groups: diazirine, alkyne, and alkaline‐cleavable alkyl ester moieties. An archaeal pyrrolysyl‐tRNA synthetase was engineered to incorporate DiZAAsu efficiently into proteins in mammalian cells. We demonstrated the in‐cell photoreactive function of diazirine by cross‐linking the DiZAAsu‐introduced GRB2 protein to its binding partner, SHC. Using the alkyne group for biotinylation, we established a tandem affinity purification strategy that enabled efficient enrichment of the cross–linked complex, thereby reducing nonspecific protein contamination. The alkaline‐based cleavage of the ester group in DiZAAsu was also demonstrated, confirming its potential for the dissociation of covalently linked complexes. This system thus expands the design space of multifunctional PAAs and adds alkaline‐based dissociation to the limited repertoire of available cleavage strategies.

## Graphical abstract

A novel noncanonical amino acid, DiZAAsu, possessing photo‐cross‐linking, biotinylation, and alkaline‐cleavable functionalities, is genetically encoded by an engineered pyrrolyl‐tRNA synthetase mutant. The alkyl ester moiety expands the design space of cleavable photo‐cross‐linkable amino acids.

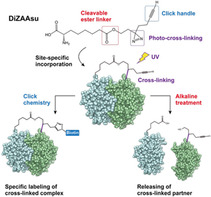

## 1 Introduction

In‐cell protein photo‐cross‐linking methods using genetically encoded photo‐cross‐linkers have provided a powerful approach to investigating direct protein–protein interactions in living cells by rapidly and covalently stabilizing the protein complexes upon light exposure [1, 2, 3]. A variety of photo‐cross‐linkable amino acids (PAAs) with different photoreactive groups, linker lengths, and chemical handles for additional modification have been encoded into the UAG codon by using the pair of UAG‐decoding tRNA and its cognate aminoacyl‐tRNA synthetase (aaRS), designed specifically for the amino acid. These PAAs are site‐specifically incorporated into a protein of interest (POI) [4, 5, 6, 7]. Once expressed in a living cell, the photo‐cross‐linkable POI can covalently capture its binding partners (BPs) upon light irradiation; the cross‐linking results likely reflect the nature of PPIs in the cellular context. In addition, the timing of the cross‐linking reaction can be controlled by light irradiation, and the site‐specific incorporation of PAA has less effect on the structure of POIs than fusion proteins with specific domains. These features differ substantially from those of conventional proximity labeling techniques such as BioID and TurboID [8, 9], which require the fusion of a large, function‐perturbing enzyme domain and label proteins based on proximity, capturing indirect interactors rather than only direct physical binding partners.

The cross‐linked complexes are typically affinity‐purified and then analyzed by mass spectrometry. Site‐specific incorporation of PAAs into POIs enables identification of their endogenous interacting proteins [4, 10, 11]. However, identifying true cross‐linked partners of the POI is often hampered by co‐purified abundant nonspecific proteins. Furthermore, the determination of cross‐linking sites that offer detailed information on the PPI interface can be challenging because a sterically hindered environment around the cross‐linking site prevents the approach of the peptidase, and the resulting comparatively high molecular weight peptides reduce detection sensitivity in mass spectrometry.

To address this limitation, a series of cleavable PAAs containing a labile selenium (Se)–carbon (Se–C) bond in the linker structure has been developed [11, 12, 13]. These linkers have proven effective for applications such as BP enrichment and interaction interface mapping via tag‐transfer. For example, DiZASeC, which has a terminal alkyne tag capable of labeling with biotin, allows for enrichment of BPs from nonspecifically bound proteins by cross‐linking and releasing them and for the identification of endogenous substrates of enzymes in *Escherichia coli* and mammalian cells [14, 15]. DiZHSeC has been used to determine the interaction interface between POI and BP, where a remnant of the linker transferred to the cross‐linked BP peptides acts as an “MS‐label” and facilitates the mapping of the cross‐linked sites by tracing the MS‐labeled peptides in mass spectrometry [16]. However, the widespread adoption of these PAAs has been limited, likely due to the challenges associated with their chemical synthesis, which often involves toxic and unstable selenium reagents.

In this study, we designed and synthesized a novel class of trifunctional PAAs based on a simple and stable alkyl ester linker. These PAAs, equipped with functionalities for photo‐cross‐linking, biotin labeling, and mild alkaline cleavage, were site‐specifically incorporated into a POI using a newly engineered *Methanosarcina mazei* pyrrolysine‐tRNA synthetase (*Mm*PylRS) in mammalian cells. To assess whether the PAAs could exert their expected functions, we introduced one of the PAAs, DiZAAsu, into growth factor receptor‐bound protein 2 (GRB2) in mammalian cells and successfully demonstrated the photo‐cross‐linking with its known BP, SHC‐transforming protein (SHC), and specific biotinylation of the cross‐linked product. Furthermore, we demonstrated the inherent cleavability of the alkyl ester linker under mild alkaline conditions, confirming its potential for dissociating covalently linked complexes. Thus, this system expands the design space of multifunctional PAAs and the repertoire of available cleavage strategies.

## 2 Results and Discussion

### 2.1 In Silico Assessment of the Recognition Potential of an Alkyl Ester Amino Acid by a *Mm*PylRS Mutant

**FIGURE 1:**
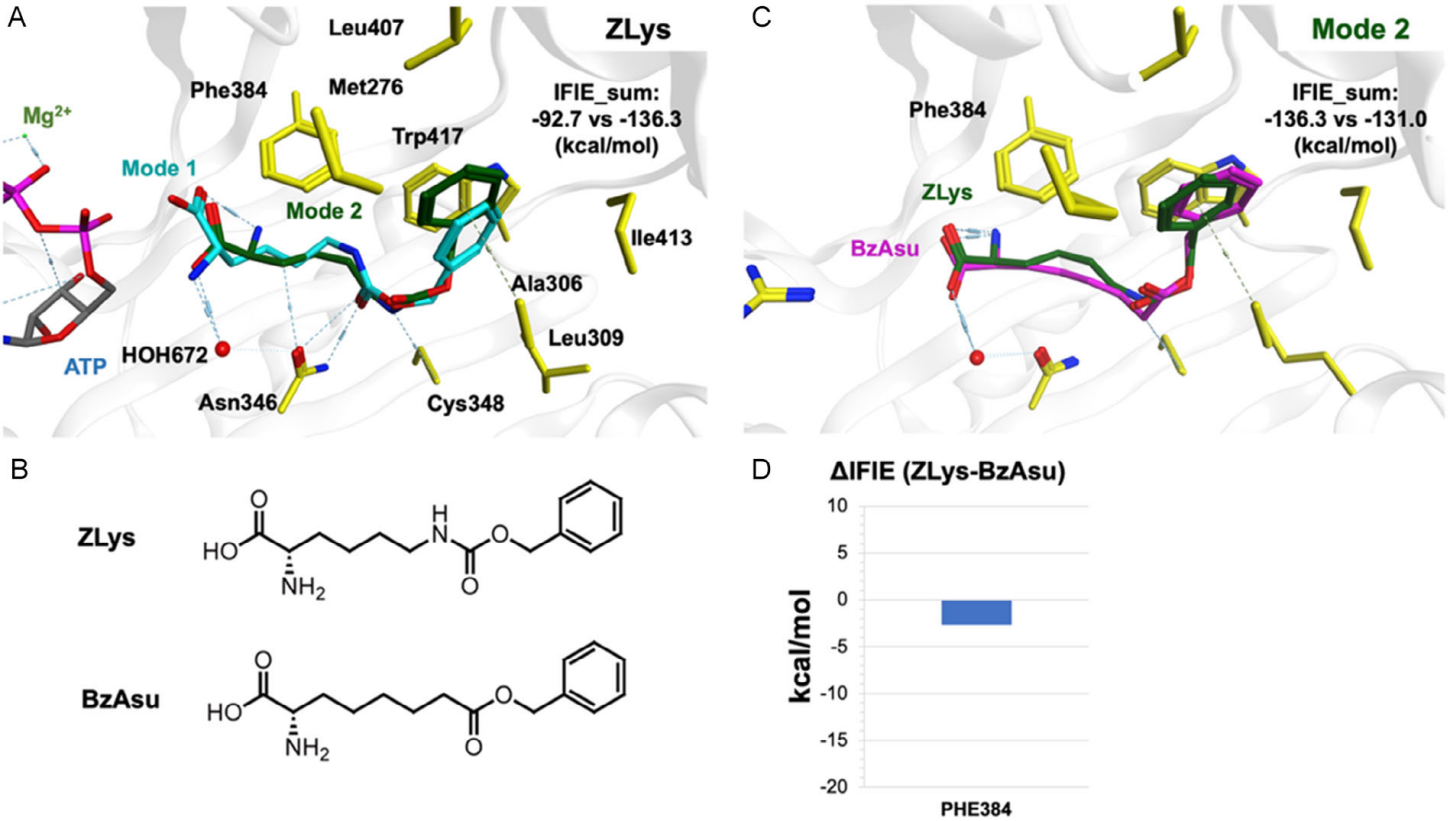
Modeling of the BzAsu‐*Mm*PylRS mutant complex and assessment of ligand‐protein binding energy. (A) A template structure of the ZLys‐*Mm*PylRS complex (PDB ID: 6AB8) utilized for molecular modeling. Two distinct binding modes of ZLys are shown. The binding energies (IFIE_sum) for both modes were calculated using the Fragment Molecular Orbital method (FMODB IDs: M2Z9Z and 92QM2). Mode 2 exhibits greater potency compared to Mode 1. (B) The chemical structures of ZLys and BzAsu are depicted, highlighting their structural differences in the linker (an N*ε*‐amine versus a methylene group). (C) Comparison between the BzAsu model and ZLys reveals a slight variation in the carboxy linker's proximity to Phe384, indicating a modification in their CH‐*π* interactions. An overall difference of 5.3 kcal/mol in the IFIE_sum values was detected (FMODB IDs: L6R79 and 34G7L). (D) Phe384 was identified as a residue primarily contributing to the observed differences in inter–fragment interaction energies.

We previously developed a *Mm*PylRS Y306A/Y384F mutant capable of recognizing a variety of structurally distinct lysine derivative noncanonical amino acids (ncAAs) that share a common carbamate group (‐NH‐CO‐O‐) containing an N*ε* atom [17]. Analysis of the 3D binding modes between the *Mm*PylRS mutant and lysine derivatives, based on their co‐crystal structure, demonstrated that this enzyme primarily recognizes the carbonyl oxygen rather than the amino group of the carbamate group (Figure 1A). Based on these findings, we hypothesized that instead of the carbamate group, ncAAs containing an alkyl ester group (‐CH_2_‐CO‐O‐), forming an alkaline‐cleavable linker structure, could also be recognized by *Mm*PylRS. To test this hypothesis, ZLys and ω‐benzyl 2‐amino‐l‐suberic acid (designated as BzAsu) were selected as model amino acids differing only in their carbamate versus alkyl ester groups (Figure 1B).

The Fragment Molecular Orbital (FMO) method was used to evaluate the binding affinity of these compounds to the *Mm*PylRS mutant. Since ZLys exhibits two distinct binding modes with *Mm*PylRS [17], we first compared the ligand‐protein inter–fragment interaction energy (IFIE_sum) of the two modes. Our calculations revealed that Mode 2 exhibited a stronger binding affinity than Mode 1 (−136.3 kcal/mol vs. −92.7 kcal/mol) (Figure 1A). Therefore, we decided to focus on Mode 2 in the subsequent analysis. The ligand‐protein and residue‐specific IFIEs were calculated and compared with ZLys based on a corresponding model of Mode 2 for BzAsu (Figure 1C). Only a slight weakening in binding energy (5.3 kcal/mol overall) was observed for BzAsu compared to ZLys when bound to the *Mm*PylRS mutant (Figure 1C,D). This slight discrepancy may primarily result from reduced CH‐*π* interactions between the benzyl group of Phe384 and the carbon linker of BzAsu, potentially influenced by changes in the local charge environment due to substituting an amine group with a methylene group. These findings suggest that ncAAs containing an alkyl ester group, such as 2‐amino‐l‐suberic acid (Asu) derivatives, may still be recognized by the *Mm*PylRS mutant and genetically encoded.

### 2.2 Site‐Specific Incorporation of Asu Derivatives into EGFP in Mammalian Cells

**FIGURE 2:**
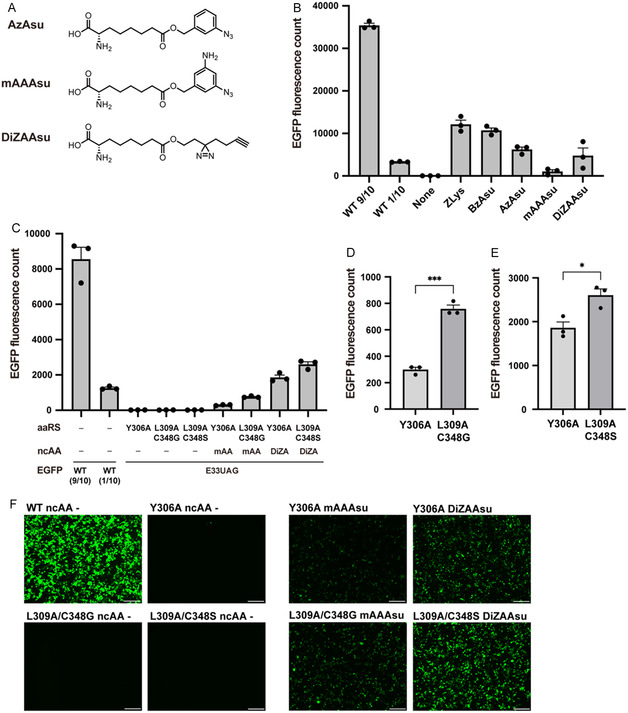
Site‐specific incorporation of newly designed ncAAs into EGFP with a series of aaRS variants. (A) Structures of AzAsu, mAAAsu, and DiZAAsu. (B) Expression of the full‐length EGFP(E33UAG) in 293 c18 cells. Total fluorescence counts from the lysates of the cells expressing the *Mm*PylRS Y306A/Y384F mutant and tRNA^Pyl^ in the presence and absence of 0.1 mM of the indicated ncAAs. To confirm that the fluorescence signal was within the linear range, the lysate from wild‐type EGFP‐expressing cells was divided into a 9:1 ratio (WT 9/10 and WT 1/10, respectively), and their fluorescence was measured. (C) Total fluorescence counts from the lysates of the cells expressing the screened mutants of *Mm*PylRS and tRNA^Pyl^ in the presence and absence of 0.1 mM of the indicated ncAAs. mAA and DiZA represent mAAAsu and DiZAAsu, respectively. For simplicity, the Y384F mutation common to all *Mm*PylRS variants is omitted from the notation. (D,E) Comparison of ncAA incorporation efficiencies when using the conventional (Y306A) and the newly screened (L309A/C348G and L309A/C348S) *Mm*PylRS mutants for mAAAsu (D) and DiZAAsu (E). (F) Representative fluorescence microscopy images of 293 c18 cells expressing full‐length EGFP dependent on the *Mm*PylRS mutants and ncAAs. Scale bar, 200 µm. The values represent mean ± SE (*n* = 3). *p < 0.05, ***p < 0.001, *p*‐values were calculated using an unpaired Student's t‐test.

The promising results from our in silico analysis encouraged us to examine the incorporation of Asu derivatives into proteins in mammalian cells. We synthesized three new Asu‐derivative PAAs designated as AzAsu, mAAAsu, and DiZAAsu. AzAsu and mAAAsu have a phenyl azide photoreactive group, and the latter has an additional amine on the benzene ring that serves as a chemical handle for further modification [5]. DiZAAsu has an alkyl diazirine photoreactive group and a terminal alkyne for modification through the “click” chemistry (Figure 2A) [18]. Notably, these amino acids were synthesized via a straightforward dehydration condensation of the corresponding alcohols and alpha‐amine‐protected Asu, followed by deprotection (Scheme S1–S3).

We then examined whether these Asu derivatives could be incorporated into proteins in mammalian cells. To assess incorporation, 293 c18 cells were transfected with plasmids encoding the *Mm*PylRS Y306A/Y384F mutant, a UAG‐decoding *Mm*tRNA^Pyl^, and EGFP harboring a TAG codon at position 33. Cells were then cultured in media containing the ncAAs. Full‐length EGFP expression and fluorescence indicated successful incorporation of ncAAs at the TAG site. The fluorescence intensities from the lysates of cells supplemented with either BzAsu or ZLys were almost equivalent, suggesting that the *Mm*PylRS Y306A/Y384F mutant could recognize an Asu derivative amino acid as well as a previously used Lys derivative, consistent with our in silico simulation (Figure 1). When AzAsu, mAAAsu, and DiZAAsu were supplemented to the cells, the fluorescence was also detected with higher intensities than the no‐ncAA control, suggesting the successful incorporation of these Asu‐based PAAs at the TAG codon‐specified site of EGFP. However, the incorporation efficiency of these PAAs was significantly lower than that of BzAsu (Figure 2B), probably reflecting a suboptimal fit within the *Mm*PylRS Y306A/Y384F substrate binding pocket.

We then aimed to develop *Mm*PylRS mutants that could introduce Asu‐based PAAs into proteins more efficiently. Through an extensive review of previous reports, we found that many *M. mazei* and *M. barkeri* PylRS mutants capable of encoding various ncAAs commonly adopted amino acid substitutions, including Y306A, L309A, C348X (where X = any amino acid), and Y384F in *Mm*PylRS, and the corresponding Y271A, L274A, C313X, and Y349F in *Mb*PylRS, respectively (Table S1). Therefore, we constructed a focused library of *Mm*PylRS mutants possessing combinations of these key substitutions and then performed a small‐scale screening with the expression of EGFP as an indicator (Figure S1). Several *Mm*PylRS mutants that introduced PAAs into proteins more efficiently than *Mm*PylRS Y306A/Y384F were identified. Among them, the L309A/C348G/Y384F and the L309A/C348S/Y384F mutants were optimal for the incorporation of mAAAsu and DiZAAsu, respectively (Figure S1). Using their optimized *Mm*PylRS mutants, the incorporation efficiencies of DiZAAsu and mAAAsu were significantly improved compared to those obtained with the conventional Y306A/Y384F mutant, as shown by total fluorescent count (Figure 2C–E) and cellular fluorescent imaging (Figure 2F). To ensure accurate comparison of the incorporation efficiencies between the newly screened and conventional mutants, we normalized the fluorescence intensities in Figure 2D,E to the amounts of two prominent proteins in the cell lysates (Figure S2). This analysis independently confirmed that the incorporation efficiencies of mAAAsu and DiZAAsu were significantly higher using the optimized *Mm*PylRS mutants compared to the conventional mutant. The site‐specific incorporation of these Asu‐based PAAs at position 33 of EGFP with their optimized *Mm*PylRS mutants was confirmed by mass spectrometry (Figure S3). Notably, while Asu‐containing peptides derived from the degradation of AzAsu and mAAAsu (and to a lesser extent, BzAsu) were detected in the MS1 data, the alkyl ester linker of DiZAAsu was found to be comparably stable. The difference in Asu production may be due to differences in substrate recognition by esterases or susceptibility to degradation during the mass spectrometry; benzyl esters (AzAsu, mAAAsu, BzAsu) are prone to fragmentation during ionization to form stable benzyl cations, whereas the alkyl ester of DiZAAsu is resistant to such cleavage. These observations support the conclusion that DiZAAsu is successfully incorporated and maintained stably within the cellular environment.

These results demonstrate that a focused library of limited mutations in key amino acid residues can efficiently identify *Mm*PylRS mutants that recognize new amino acids through single‐round screening. The *Mm*PylRS(L309A/C348S/Y384F) mutant for DiZAAsu is homologous to the *Mb*PylRS mutant used to encode Se‐containing DiZASeC (compound **1** in Table S1). The two amino acids have a highly similar overall structure, with the key difference being that DiZAAsu has an ester (‐CH_2_‐CO‐O‐) and DiZASeC has an amide bond (‐CH_2_‐CO‐NH‐), at the position recognized by PylRS. This finding confirms that PylRS primarily recognizes the carbonyl atom, independent of variations in the adjacent chemical groups. In subsequent studies, we used *Mm*PylRS Y306A/Y384F, L309A/C348G/Y384F, and L309A/C348S/Y384F mutants for AzAsu, mAAAsu, and DiZAAsu, respectively.

### 2.3 Photo‐Cross‐Linking of the Proteins Containing Asu‐Based PAAs in Mammalian Cells

**FIGURE 3:**
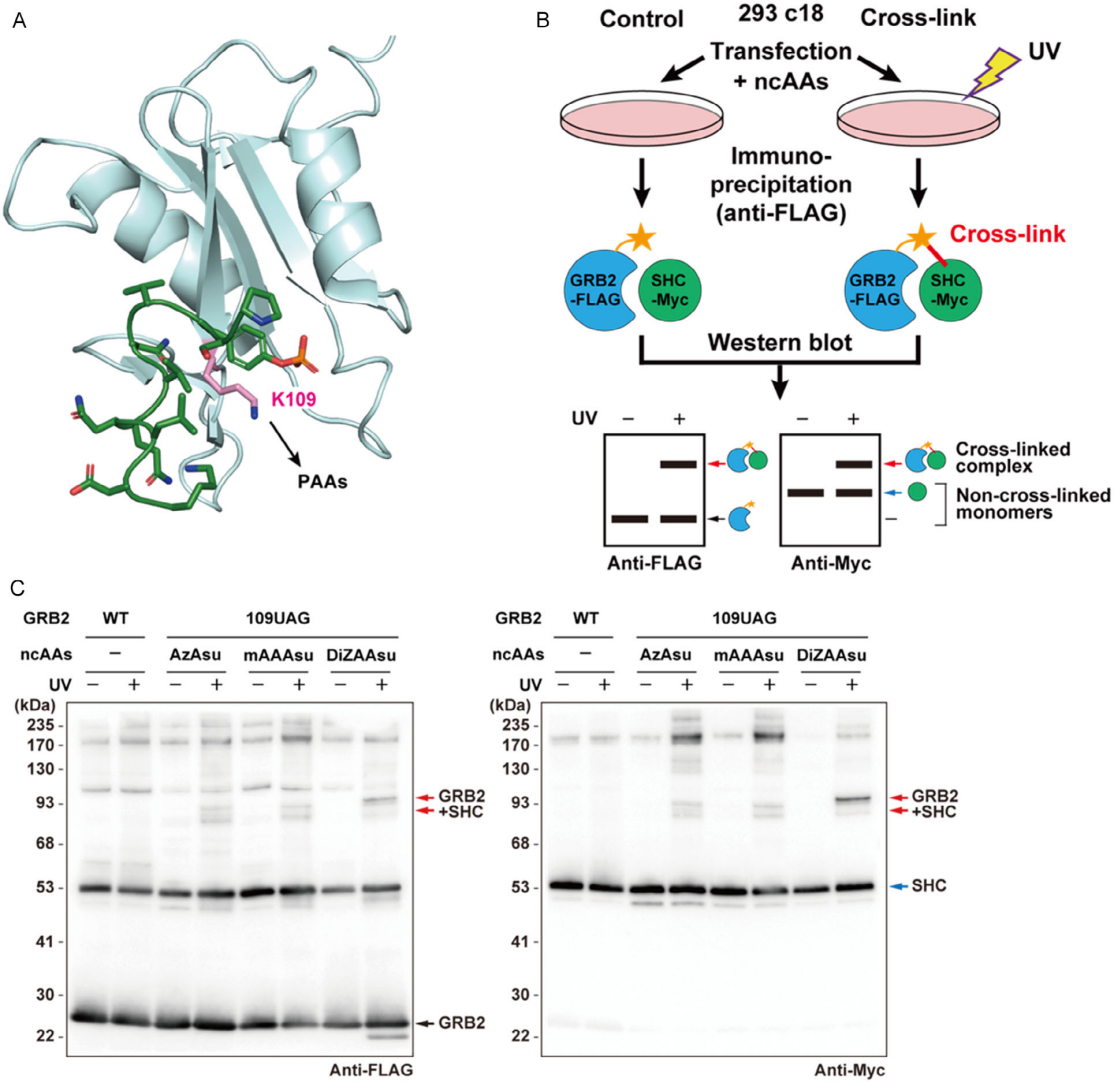
Cross‐linking of the PAA‐incorporated GRB2 mutants with SHC in 293 c18 cells. (A) 3D structure of the SH2 domain of GRB2 (cyan) and the phosphotyrosine‐containing peptide (green) corresponding to SHC (315–325) (PDB ID: 1QG1). GRB2 K109 residue, the site for PAA incorporation, is shown in pink. (B) Schematic illustration of in‐cell protein cross‐linking between GRB2‐FLAG and SHC‐Myc, followed by immunoprecipitation and western blot analysis. A UV‐dependent cross‐linked product is detected as a higher molecular weight band on the blot in addition to the noncross‐linked GRB2‐FLAG and noncovalently co‐purified SHC monomer proteins. (Adapted from Noguchi Y. et al., 2019, *Development* [23] doi: 10.1242/dev.163618, with permission from The Company of Biologists). (C) Photo‐cross‐linking of GRB2‐FLAG mutants containing the indicated PAAs at position 109 with SHC‐Myc. The 293 c18 cells expressing wild‐type GRB2‐FLAG or its PAA‐containing mutants together with SHC‐Myc were irradiated with UV‐A (365 nm) for 15 min on ice. Protein complexes, including GRB2‐FLAG, were affinity‐purified from cell lysates using anti‐FLAG affinity beads and analyzed by western blotting with anti‐FLAG (left panel) and anti‐Myc (right panel) antibodies. The arrows shown in red, blue, and black indicate cross‐linked GRB2–SHC dimer, SHC monomer, and GRB2 monomer, respectively. The band around 53 kDa in the anti‐FLAG blot (left panel) corresponds to the overexpressed SHC protein nonspecifically detected by the antibody.

To assess the photo‐cross‐linking capabilities of the newly synthesized Asu‐based PAAs, we conducted validation studies using a model protein interaction pair. It has been reported that GRB2 interacts with phosphorylated SHC following epidermal growth factor (EGF) stimulation [19, 20]. In addition, our previous studies demonstrated that incorporating various PAAs at position 109 of GRB2 enables efficient cross‐linking with SHC in mammalian cells [10, 21, 22]. Therefore, the cross‐linking function of the three Asu‐based PAAs was evaluated at this established position using the pair of GRB2 (POI) and SHC (BP) as a model PPI. The GRB2 protein containing AzAsu, mAAAsu, or DiZAAsu at position 109 with a C‐terminal FLAG‐tag was expressed together with Myc‐tagged SHC (p52 isoform) and a low level of EGF receptor (EGFR) in 293 c18 cells (Figure 3A,B). After the EGF stimulation, the cells were irradiated with UV‐A light. The GRB2 proteins and their complexes were purified from cell lysates using anti‐FLAG affinity beads and then detected by western blotting with anti‐FLAG and anti‐Myc antibodies (Figure 3C). Western blot analysis with an anti‐FLAG antibody showed that all PAA‐incorporated GRB2 proteins, but not the wild‐type, yielded a product migrated around 90 kDa in addition to the GRB2 monomer, but only upon UV irradiation (Figure 3C, left panel). This 90 kDa band was also detected by the anti‐Myc antibody, which also recognized the SHC monomer (Figure 3C, right panel). These results indicate the formation of cross‐linked complexes between the PAA‐incorporated GRB2 proteins and SHC. Since the cross‐linked product was most clearly detected when using DiZAAsu (Figure 3C), we selected this amino acid for subsequent studies.

### 2.4 Enrichment of Cross‐Linked Proteins via Bioorthogonal Biotinylation at the Alkyne Handle of DiZAAsu

**FIGURE 4:**
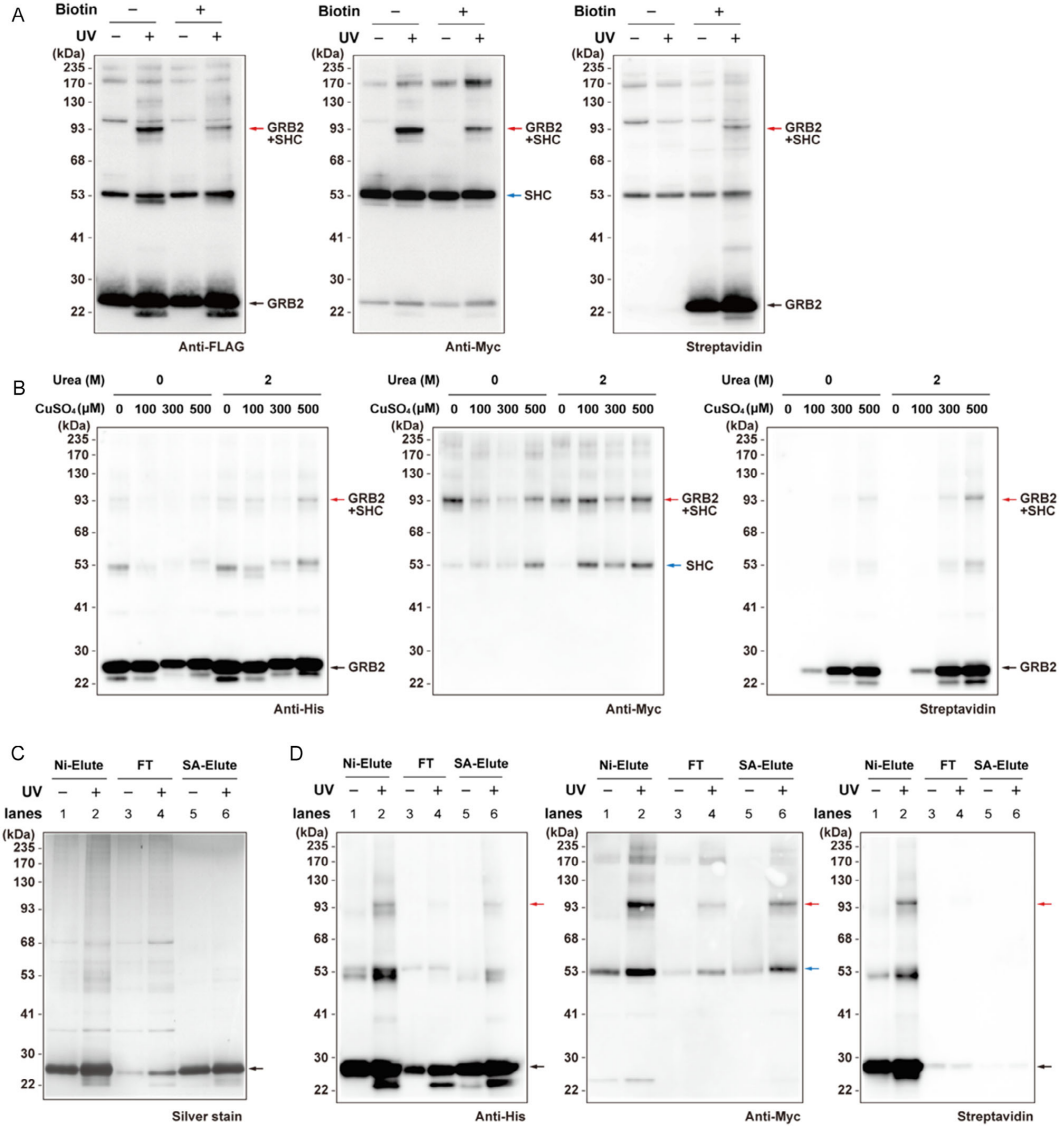
Specific biotinylation of DiZAAsu‐containing GRB2 and its cross‐linked product. (A) Biotin labeling of DiZAAsu‐containing GRB2 and its cross‐linked product. Photo‐cross‐linking between GRB2‐FLAG containing DiZAAsu at position 109 and SHC‐Myc in 293 c18 cells was performed as described for Figure 3. In the cell lysate, GRB2‐FLAG and its cross‐linked product were labeled with biotin picolyl azide via Cu‐catalyzed click chemistry, purified using anti‐FLAG affinity beads, and analyzed by western blotting with anti‐FLAG (left panel), anti‐Myc (middle panel), and Streptavidin (right panel). (B) Biotin labeling under mildly denaturing conditions. Photo‐cross‐linking between GRB2‐His containing DiZAAsu at position 109 and SHC‐Myc in 293 c18 cells was performed as described for Figure 3. Biotin labeling in cell lysates was performed in the presence or absence of 2 M urea and the indicated concentrations of CuSO_4_. GRB2‐His and its cross‐linked product were affinity‐purified using Ni‐NTA beads and analyzed by western blotting with anti‐His (left panel), anti‐Myc (middle panel), and Streptavidin (right panel). (C,D) Tandem purification of GRB2 and its cross‐linked complexes labeled with biotin using Ni‐NTA and streptavidin beads, sequentially. GRB2(109DiZAAsu)‐His and its cross‐linked products were labeled with Dde‐biotin‐picolylazide in cell lysates under the optimized conditions in (B), followed by purification using Ni‐NTA beads. The eluate from the beads (indicated as Ni‐Elute) was further incubated with streptavidin beads. The flow‐through (FT) and eluate with 2% hydrazine (SA‐Elute), together with the Ni‐Elute, were analyzed by silver staining (C) and western blotting (D). Samples corresponding to an equal volume of the initial lysates were loaded in each lane. The arrows shown in red, blue, and black indicate cross‐linked GRB2–SHC dimer, SHC monomer, and GRB2 monomer, respectively.

DiZAAsu has an alkyne handle that can be labeled with azide‐containing compounds via click chemistry. Therefore, we examined the biotinylation of a DiZAAsu‐containing protein and its cross‐linked products for their further purification with streptavidin beads. Following the same procedure described in Figure 3, DiZAAsu‐incorporated GRB2‐FLAG and SHC‐Myc were overexpressed in 293 c18 cells, and the cell lysates were collected after UV‐irradiation. Subsequently, the alkyne handle of DiZAAsu was chemically coupled to biotin‐picolyl azide using Cu(I)‐catalyzed click chemistry. The GRB2 protein and its cross‐linked products were purified with the anti‐FLAG affinity beads and analyzed by western blotting. Detection with HRP‐conjugated streptavidin confirmed that both GRB2 and its cross‐linked product with SHC were biotinylated, but only after the click chemistry reaction was performed on lysates from UV‐irradiated cells (Figure 4A, right panel). However, the amount of purified cross‐linked product was decreased after the biotinylation reaction (Figure 4A left and middle panels), probably because the proteins were oxidized and partially aggregated by the Cu(I) [24, 25] used in the click chemistry reaction, which prevented efficient purification. Indeed, the purified amount of biotinylated cross‐linked product was improved by performing the click chemistry reaction using 500 µM of CuSO_4_ under mildly denaturing conditions with 2 M urea (Figure 4B).

We further examined whether the specific biotinylation of DiZAAsu could enrich the cross‐linked complexes of GRB2 by an additional purification using streptavidin beads. In this experimental setting, we used a biotin‐picolyl azide reagent harboring a Dde linker, which is cleavable by hydrazine treatment and enables specific release of biotin‐labeled proteins from streptavidin beads. The cross‐linked product between GRB2‐His and SHC‐Myc was labeled with biotin in the cell lysates under the optimized conditions (Figure 4B) and purified in tandem using Ni‐NTA beads and then streptavidin beads. The biotin‐labeled proteins were released from the beads with 2% hydrazine solution and analyzed by silver staining and western blotting (Figure 4C). As shown in Figure 4C, nonspecific, contaminating proteins derived from the cell lysates were still included in the eluate fractions from the Ni‐NTA beads (lanes 1 and 2). After the subsequent purification using streptavidin beads, the majority of these background proteins were removed into the flow‐through (FT) fractions (lanes 3 and 4), and the final eluate fractions (SA‐Elute, lanes 5 and 6) primarily contained the bands corresponding to the GRB2 protein. In the western blotting, the Ni‐Elute fractions derived from the UV‐irradiated cell lysate (lane 2) contained the biotinylated GRB2‐His monomer and its cross‐linked product with SHC. These biotinylated proteins were efficiently captured by the streptavidin beads, as evidenced by their decrease in the FT fraction (lane 4). Both the GRB2‐His monomer and the cross–linked complex were successfully recovered in the SA‐Elute fraction, as detected by the anti‐His (left panel) and anti‐Myc (middle panel) antibodies (lane 6). Notably, these bands were absent in the streptavidin blot (right panel, lane 6). This absence confirms that the Dde‐linker was efficiently cleaved by hydrazine, releasing the proteins from the biotin tag, which remained bound to the beads, and thereby specifically eluting the target proteins. Taken together, the results from silver staining and western blotting clearly demonstrate that this tandem affinity purification strategy effectively eliminates abundant, nonspecific background proteins while specifically enriching the covalently cross–linked complex. This highlights the utility of the bioorthogonal alkyne handle on DiZAAsu for the high‐purity purification of cross‐linked PPIs.

### 2.5 Reversibility of the DiZAAsu‐Mediated Cross‐Link Under Alkaline Conditions

**FIGURE 5:**
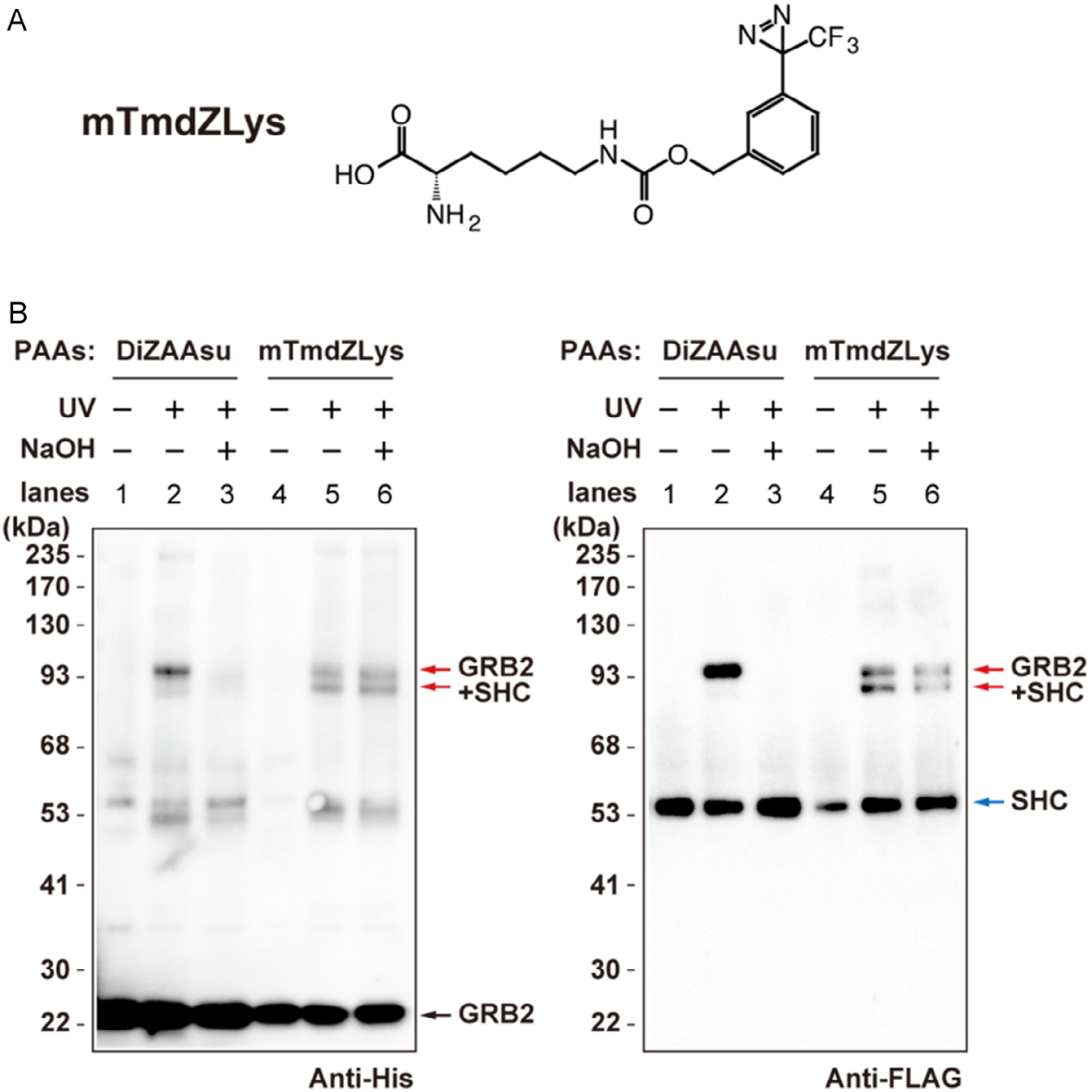
Dissociation of cross‐linked product between GRB2 and SHC by cleaving the ester linker of DiZAAsu. (A) Structure of mTmdZLys, which is a noncleavable, carbamate‐type PAA. (B) Photo‐cross‐linking between GRB2‐His containing DiZAAsu or mTmdZLys at position 109 and SHC‐FLAG in 293 c18 cells was similarly performed as described for Figure 3. GRB2‐His and its cross‐linked product were purified using Ni‐NTA beads and then incubated with or without 0.25 M NaOH at 25°C for 30 min. After neutralization, the samples were subjected to western blotting with anti‐His (left panel) and anti‐FLAG (right panel). The arrows shown in red, blue, and black indicate cross‐linked GRB2–SHC dimer, SHC monomer, and GRB2 monomer, respectively.

We finally assessed whether the covalent bond formed by the DiZAAsu cross‐linker could be reversed by cleaving the alkyl ester linker under aqueous alkaline conditions. The DiZAAsu‐incorporated GRB2‐His was co‐expressed with SHC‐FLAG in 293 c18 cells. As a direct comparison, a GRB2‐His mutant containing mTmdZLys (Figure 5A) [21], a conventional noncleavable carbamate‐type PAA, was co‐expressed with SHC‐FLAG. Following UV‐irradiation, the GRB2 proteins and their cross‐linked products were purified from the cell lysates using Ni‐NTA beads. The eluted proteins from the beads were treated with 0.25 M NaOH for 30 min and then analyzed by western blotting with anti‐His and anti‐FLAG antibodies (Figure 5B). The signal for the cross–linked complex with DiZAAsu was eliminated by the NaOH treatment (lanes 2 vs 3). In contrast, the cross‐linked product with the noncleavable mTmdZLys remained largely stable under the same treatment (lanes 5 vs 6). Importantly, the NaOH treatment did not reduce the signal of the GRB2 and SHC protein monomers (lanes 2 vs 3 and lanes 5 vs 6), ruling out the possibility of nonspecific protein degradation under these conditions. These results strongly suggest that the alkyl ester linker of DiZAAsu is specifically cleaved by alkaline treatment. Notably, although the amount of cross‐linked GRB2–SHC complexes decreased with alkaline treatment, the amount of dissociated monomeric GRB2 and SHC did not increase proportionally. This is probably due to the low amount of GRB2 and SHC derived from the cross‐linked complexes compared with the amount of overexpressed GRB2 and the SHC monomers co‐purified independently of cross‐linking.

## 3 Conclusion

In this study, our in silico analysis first suggested the feasibility of *Mm*PylRS recognizing Asu‐derivative ncAAs, which possess an alkyl ester group instead of the carbamate group found in Lys derivatives. Based on this finding, we designed and synthesized new trifunctional photo‐cross‐linkers containing a photoreactive, a chemical handle, and a cleavable alkyl ester group. Among them, DiZAAsu was incorporated into POIs with high efficiency using the *Mm*PylRS L309A/C348S mutant obtained through screening of the focused C348X library. Furthermore, we demonstrated that DiZAAsu introduced into the POI (GRB2) exerted the three designed functions: 1) in‐cell cross‐linking with its BP (SHC), 2) specific biotin labeling of the cross‐linked product via the alkyne handle, and 3) dissociating the cross‐linked product by the cleavage of the alkyl ester structure by alkaline treatment.

DiZAAsu has the same trifunctionality as the previously developed DiZASeC, as both contain a photo‐cross‐linkable diazirine, a terminal alkyne handle, and a cleavable linkage; however, they differ in the cleavable structure (Se–C and alkyl ester bonds, respectively) and thereby in the cleavage conditions. One of the advantages of DiZAAsu is that it can be straightforwardly synthesized via dehydration condensation of nonhazardous alcohols and alpha‐amine‐protected Asu, thereby increasing accessibility to the technique. In addition, whether the conditions for cleaving the Se–C bond of DiZASeC (e.g., 7 mM H_2_O_2_) or the conditions for cleaving the carbonyl ester group of DiZAAsu (e.g., 0.25 M NaOH) have smaller effects on the protein structure depends on the properties of the protein; thus, appropriate conditions must be adopted. Particularly, in the case of proteins containing many amino acids that are easily oxidized, cleavage by alkaline treatment may be more suitable. Indeed, similar alkaline conditions (e.g., 0.125 M NaOH) have been previously validated for the cleavage of ester bonds engineered into the protein backbone itself [26], supporting its utility as an acceptable condition for protein manipulation. Conversely, protein stability is highly sequence‐dependent, and high pH environments can affect ionic bonds and electrostatic interactions, potentially leading to denaturation [27]. Specific residues, such as asparagine (particularly within Asn‐Gly motifs), are also prone to alkaline‐induced deamidation via a succinimide intermediate [28, 29]. Therefore, optimizing the treatment time by monitoring the dissociation of cross‐linked products is recommended (Figure 5). Furthermore, subsequent mass spectrometry analysis should account for potential chemical modifications, such as the conversion of asparagine to aspartate. Although further optimization is required to apply DiZAAsu for the mass spectrometric mapping of interaction interfaces and the identification of endogenous BPs, the inherent cleavability of the alkyl ester moiety offers new options for designing multifunctional PAAs and for the high‐confidence analysis of direct protein interaction partners in living cells.

## 4 Experimental Section

### 4.1 Construction of the BzAsu Binding Model

A co‐crystal structure of ZLys bound to the *Mm*PylRS Y306A/Y384F mutant was obtained from the Protein Data Bank (PDB ID: 6AB8). Solvent molecules and all small ligands except ATP, Mg^2+^ ions, and HOH672 were removed (Figure 1A). ZLys exhibits two distinct binding modes in the structure, designated as modes 1 and 2, with occupancies of 0.58 and 0.37, respectively. To analyze these binding interactions separately, we divided the structure into two distinct complexes based on each binding mode and proceeded with structural refinement steps. Using the Structure Preparation and Protonate 3D modules in MOE (version 2022.02), hydrogen atoms were added, and missing residues and protonation states were fixed. We then minimized hydrogen atoms and side chain atoms with a tether constraint of 0.5 under the AMBER10:EHT force field. Finally, using the Builder module, BzAsu models were generated by substituting the *Nε*‐amine group of ZLys with a methylene (‐CH_2_‐) group. These modified BzAsu molecules were subsequently energy‐minimized within each of the two complexes to achieve stable configurations.

### 4.2 Calculation of BzAsu Binding Energies

To calculate the binding energy between BzAsu and the *Mm*PylRS Y306A/Y384F mutant and compare it with that of ZLys, we carried out Fragment Molecular Orbital (FMO) calculations. The FMO method is a widely used approach in quantum mechanics (QM) for analyzing biological molecules and their interactions with ligands or inhibitors [30]. The comprehensive methodology has been well‐documented in previous studies [31, 32, 33]. Briefly, the FMO method involves dividing large biosystems into small fragments—typically at amide bonds between amino acids—and performing ab initio orbital calculations on these fragments. This allows us to obtain the energies of all fragment monomers and dimers (Equation (1)).

(1) $$E_{\text{total}}=\sum_{i}E_{i}^{\prime }+\sum_{\mathrm{ij}}\text{Δ}{\tilde{E}}_{\mathrm{ij}}$$

Here, $E_{i}^{\prime }$ represents the energy of the fragment monomer, and $\Delta {\tilde{E}}_{ij}$ (Equation (1)) denotes the inter–fragment interaction energy. To calculate the binding energy between a ligand and a receptor, we set i=ligand fragment and j∈receptor fragments. The total binding energy is then expressed as IFIE_sum (Equation (2)).

(2) $$\begin{array}{c} \text{IFIE}\_\mathrm{sum}=\sum_{\begin{array}{c} i=\text{ligand fragment},\\ j\in \text{receptor framents} \end{array}}\text{Δ}{\tilde{E}}_{\mathrm{ij}} \end{array}$$

In addition to calculating ligand‐receptor binding energies, we can also determine the interaction energies between the ligand and individual residues within the receptor. This approach allows us to analyze both overall binding affinity and specific residue contributions, providing deeper insights into molecular interactions. To simplify the FMO calculation, ATP and Mg^2+^ ions were removed. The FMO calculation was performed under the second‐order Møller–Plesset perturbation theory (MP2) with the 6‐31G* basis set using the ABINIT‐MP Program Development Edition (version rev23q2), and all computations were executed on the supercomputer of Fugaku.

### 4.3 Plasmids

The pOriP vector, which contains the replication origin derived from the Epstein–Barr virus, was used for high‐level gene expression in 293 c18 cells. The pOriP‐based vectors carrying the genes for the *Mm*PylRS mutants (R61K/G131E/Y384F and R61K/G131E/Y306A/Y384F, hereafter referred to as Y384F and Y306A/Y384F, respectively), and nine copies of the MmtRNA^Pyl^ expression cassette (each consisting of the human U6 promoter, MmtRNA^Pyl^, and the human U6 terminator) were described previously [34]. Separately, the pOriP‐based vectors carrying the genes for wild‐type human GRB2 and its mutant with an amber (TAG) codon at position 109, and wild‐type EGFP and its mutant with an amber (TAG) codon at position 33, each with a C‐terminal FLAG‐tag, were described previously [10, 21, 35]. The pcDNA4/TO‐based vectors for expressing human SHC p52 and human EGFR, each with a C‐terminal Myc‐tag, were also previously described [10]. The expression plasmids for C‐terminal His‐tagged GRB2 and C‐terminal FLAG‐tagged SHC were constructed as follows: the coding regions of GRB2 and SHC were amplified from the above‐mentioned pOriP and pcDNA4/TO vectors, respectively, using primers designed to add the new tag sequences. These resulting fragments were then cloned into the corresponding original vector backbones, replacing the original genes. Expression vectors for the *Mm*PylRS mutants L309A/Y384F and Y306A/L309A/Y384F were created by site‐directed mutagenesis using the pOriP‐*Mm*PylRS(Y384F) vector as a template. These newly generated vectors and the pOriP‐*Mm*PylRS(Y306A/Y384F) vector were then used as templates for a second round of mutagenesis to introduce substitutions at the C348 position. The DNA primers used for the site‐directed mutagenesis are listed in Table S2.

### 4.4 Incorporation of ncAAs into EGFP in Mammalian Cells

Cells (293 c18 [HEK293‐EBNA, RRID: CVCL_6974], American Type Culture Collection) were seeded at 2.0 × 10^5^ cells per well in a 24‐well plate pre‐coated with poly‐d‐lysine (PDL; BD Biosciences, San Jose, CA, USA) and cultured in Dulbecco's Modified Eagle's Medium (DMEM) high glucose (Nacalai Tesque, Kyoto, Japan) supplemented with 10% (v/v) heat‐inactivated FBS (Sigma–Aldrich, St. Louis, MO, USA) for 24 h at 37°C in 5% CO_2_. Cells were routinely cultured to 90‐100% confluency before transfection. For the site‐specific incorporation of ncAAs at position 33 of EGFP with a C‐terminal FLAG tag, the cells were co‐transfected with the pOriP plasmids encoding EGFP (E33UAG), *Mm*tRNA^Pyl^, and *Mm*PylRS mutants using Lipofectamine 2000 in Opti‐MEM I reduced‐serum medium (Thermo Fisher Scientific, Waltham, MA, USA). The transfection medium was then replaced with fresh DMEM containing 0.1 mM of ncAAs for further incubation. In addition, cells transfected with pOriP‐EGFP(WT) alone and pOriP‐EGFP(E33UAG) alone were prepared as positive and negative controls, respectively. After a 24‐h incubation, fluorescence images of the cells were acquired using a BZ‐X800 fluorescence microscope (Keyence, Osaka, Japan). The cells were lysed in lysis buffer (50 mM HEPES [pH 7.5], 150 mM NaCl, 1% Triton X‐100, 10% glycerol, 100 mM NaF, and protease inhibitor cocktail [Roche, Basel, Switzerland]). The lysates were transferred to a 96‐well black plate to measure EGFP fluorescence at an excitation/emission of 475/500–550 nm using a GloMax Discover microplate reader (Promega, Madison, WI, USA).

### 4.5 In‐Cell Protein Photo‐Cross‐Linking of GRB2 with SHC

Cells (293 c18) were seeded at 1.6 × 10^6^ cells in 6‐cm dishes and cultured in DMEM high glucose with 10% (v/v) FBS as described above. For the expression of PAA‐containing GRB2‐FLAG, 0.75 µg, 0.5 µg, and 1.25 µg of pOriP‐based vectors encoding GRB2(109UAG)‐FLAG, *Mm*PylRS mutants, and *Mm*tRNA^Pyl^, respectively, were co‐transfected with 1.25 µg and 0.25 µg of pcDNA4/TO‐based vectors encoding SHC‐Myc and EGFR‐Myc, respectively, using Lipofectamine 2000 in Opti‐MEM I containing 0.1 mM of AzAsu, mAAAsu, or DiZAAsu, or 10 µM (nearly saturated concentration) of mTmdZLys. For the wild‐type control, the vector encoding GRB2(109UAG)‐FLAG was replaced with one for wild‐type GRB2‐FLAG, and co‐transfected with all other plasmids. After 20 h of incubation, the cells were stimulated at 37°C for 5 min with 100 ng/mL EGF in Opti‐MEM I to promote the tyrosine phosphorylation of downstream proteins. The cells were washed twice with Hanks’ Balanced Salt Solution (HBSS) and exposed to UV‐A light for 15 min on ice to form cross‐linked complexes between GRB2 and its interacting proteins, including SHC. The cross‐linking of GRB2‐His with SHC‐Myc or SHC‐FLAG was performed using the same procedure. The cells were then lysed and subjected to subsequent analyses.

### 4.6 Purification of Proteins

For the purification of FLAG‐tagged proteins, lysates of the cells prepared as above were incubated with an anti‐FLAG M2 affinity gel (Sigma–Aldrich) at 4°C for 15 min. The beads were washed twice with IP buffer (50 mM HEPES [pH 7.5], 150 mM NaCl, 1% Triton X‐100, 100 mM NaF, and protease inhibitor cocktail [Roche, Basel, Switzerland]), and proteins bound to the beads were eluted with 0.5 mg/mL of DYKDDDDK (FLAG) peptide (FUJIFILM Wako Pure Chemical Corporation, Osaka, Japan) solubilized in IP buffer. For the purification of His‐tagged proteins, the lysate of the cells was added to His Mag Sepharose Ni (Cytiva) that had been washed with PBS containing 5 mM imidazole and then incubated at 4°C for 30 min. After washing with PBS containing 5 mM imidazole and 1% SDS, the proteins were eluted with PBS containing 200 mM imidazole.

### 4.7 Silver Staining and Western Blotting

Silver staining of the proteins separated by SDS‐PAGE was performed using EzStain Silver kit (ATTO Corporation, Tokyo, Japan). For western blotting, the proteins separated by SDS‐PAGE were transferred to a PVDF membrane (Millipore, Burlington, MA, USA). The membrane was blocked with 5% (w/v) skim milk or 5% (w/v) Bovine Serum Albumin (BSA) overnight at 4°C. For protein detection, the membrane was first incubated with one of the following primary antibodies: a rabbit polyclonal anti‐DYKDDDDK (FLAG) tag antibody (1:4000 dilution; Proteintech, Rosemont, IL, USA) or a rabbit polyclonal anti‐Myc tag antibody (1:3000 dilution; Proteintech). After washing, the membrane was incubated with a horseradish peroxidase (HRP)‐conjugated goat anti‐rabbit IgG secondary antibody (1:20000 dilution; Jackson ImmunoResearch, West Grove, PA, USA). For direct detection, blots were incubated with either an HRP‐conjugated mouse monoclonal anti‐His tag antibody (1:3000 dilution; Proteintech) or HRP‐conjugated streptavidin (1:5000 dilution; Proteintech).

### 4.8 Biotin Labeling of Alkyl Ester‐Type Photo‐Cross‐Linker by Click Reaction

The alkyne handle of DiZAAsu incorporated into POIs was biotin‐labeled by a Cu(I)‐based click reaction. First, BTTAA and CuSO_4_ were mixed, to which Biotin Picolyl Azide or Dde biotin picolyl azide (both from Sigma–Aldrich) was added to make a premix. The supernatant of cell lysates containing 2 M Urea was added to the premix, mixed with ascorbic acid, and incubated at 25°C for 1 h under light‐shielded conditions. The final concentration of each reagent was prepared to be 500 μM for BTTAA, 500 μM for CuSO_4_, 1 mM for Biotin Picolyl Azide, and 2.5 mM for ascorbic acid, respectively. For the tandem purification, the biotin‐labeled proteins were affinity‐purified using Ni‐NTA and streptavidin beads sequentially. The eluate from Ni‐NTA beads was further incubated with streptavidin beads at 4°C for 1 h. After washing with PBS containing 0.5% SDS, the proteins were eluted with 2% hydrazine containing 0.5% SDS for 2 h. The tandem‐purified proteins were subjected to SDS‐PAGE, silver staining, and WB.

### 4.9 Alkyl Ester‐Type Photo‐Cross‐Linker Cleavage

For the cleavage of ester‐type photo‐cross‐linkers, the eluate from Ni‐NTA beads was incubated with 0.25 M NaOH for 30 min at 25°C. After neutralization with HCl, the proteins were subjected to SDS‐PAGE followed by WB.

### 4.10 Statistics

Statistical analyses were performed using the Prism 10 (version 10.6.0) software (GraphPad Software Inc., San Diego, CA). Significant difference was determined using unpaired Student's t‐test. *p* < 0.05 was considered statistically significant.

## Supporting Information

Additional supporting information can be found online in the Supporting Information section. **Supporting Scheme S1:** Synthesis of AzAsu **(4)**. **Supporting Scheme S2:** Synthesis of DiZAAsu **(8)**. **Supporting Scheme S3:** Synthesis of mAAAsu **(11)**. **Supporting Fig. S1:** Small‐scale screening of *M. mazei* PylRS mutants with which newly designed ncAAs are incorporated more efficiently. **Supporting Fig. S2:** Normalized comparison of PAA‐incorporation efficiencies between conventional and newly screened *Mm*PylRS mutants. **Supporting Fig. S3:** Site‐specific incorporation of Asu‐based ncAAs at position 33 of EGFP. **Supporting Table S1:** List of reported PylRS mutants. **Supporting Table S2:** List of DNA primers for site‐directed mutagenesis.

## Author Contributions

**Masahiro Takayama:** formal analysis (lead), investigation (lead), validation (equal), visualization (lead), writing – original draft (lead), writing – review and editing (equal). **Tomoya Tsubota:** formal analysis (equal), investigation (lead), validation (lead), visualization (equal), writing – original draft (equal). **Takao Yamaguchi:** funding acquisition (equal), investigation (equal), visualization (equal), writing – original draft (equal). **Kosuke Chiba:** investigation (equal). **Takumi Yoshida:** investigation (equal). **Yoshiyuki Hari:** investigation (equal), visualization (equal), writing – original draft (equal). **Yu‐Shi Tian:** investigation (equal), visualization (equal), writing – original draft (equal). **Daisuke Takaya:** investigation (supporting), writing – original draft (supporting). **Asuka Mori:** investigation (supporting), validation (equal). **Tomohito Tsukamoto:** supervision (equal), writing – review and editing (supporting). **Kenji Ishimoto:** supervision (equal), writing – review and editing (supporting). **Yukio Ago:** supervision (supporting), writing – review and editing (supporting). **Yoshiaki Okada:** supervision (supporting), writing – review and editing (supporting). **Kensaku Sakamoto:** resources (equal), supervision (supporting); writing – review and editing (equal). **Takefumi Doi:** funding acquisition (lead), supervision (equal), writing – review and editing (supporting). **Kaori Fukuzawa:** supervision (equal), writing – review and editing (supporting). **Satoshi Obika:** supervision (lead), writing – review and editing (equal). **Shinsaku Nakagawa:** supervision (lead), writing – review and editing (supporting). **Nobumasa Hino:** conceptualization (lead), data curation (lead), formal analysis (equal), funding acquisition (lead), project administration (lead), visualization (equal), writing – original draft (lead), writing – review and editing (lead).

## Conflicts of Interest

The authors declare the following competing financial interest(s): M.T. is an employee of Shionogi & Co.,Ltd. The other authors declare no conflicts of interest.

## Supporting information

Supplementary Material

## Data Availability

The FMO calculation results are deposited in FMODB (https://drugdesign.riken.jp/FMODB/) [36], and their FMODB IDs are M2Z9Z, 92QM2, L6R79, and 34G7L.
