# Supplementary material for: Genetic Encoding of a Trifunctional Photo‐Cross‐Linker with a Cleavable Alkyl Ester Moiety
Source: Chembiochem. 2026 Jan 30;27(3):e202500827. doi: 10.1002/cbic.202500827 (PMC12859177; doi:10.1002/cbic.202500827)
Supplement: Supplementary file 1 — Supplementary Material [file CBIC-27-e202500827-s001.zip › MT_Revision TableS1 submit.pdf]

Table S1 List of reported PylRS mutants

Number	Structure	PylRS spieces	Mutation														Reference
		M. barkeri		D76		M241	L266	A267	L270	**Y271**	**L274**	N311	**C313**	**Y349**	V366	W382	
		M. mazei	R61		G131		L301	A302	L305	**Y306**	**L309**	N346	**C348**	**Y384**	V401	W417	
**1**	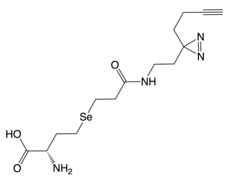	M. barkeri									A		A/S	F			1
**2**	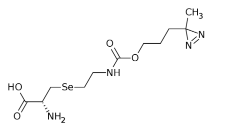	M. barkeri									A		S	F			2
**3**	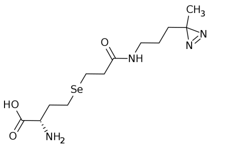	M. barkeri									A		S	F			2
**4**	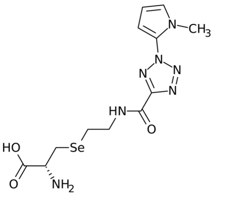	M. mazei								V	A		F	F			3,4
**5**	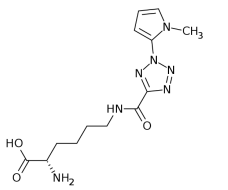	M. mazei								V	A		F	F			3
**6**	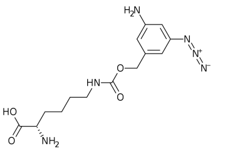	M. mazei	K		E					A				F			5
**7**	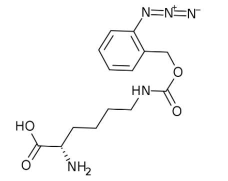	M. barkeri								A				F			6
**8**	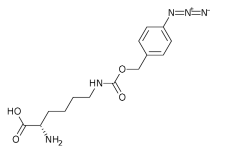	M. barkeri									A		S	F			7

Table S1 List of reported PylRS mutants (continued)

Number	Structure	PylRS spieces	Mutation														Reference
		M. barkeri		D76		M241	L266	A267	L270	**Y271**	**L274**	N311	**C313**	**Y349**	V366	W382	
		M. mazei	R61		G131		L301	A302	L305	**Y306**	**L309**	N346	**C348**	**Y384**	V401	W417	
**9**	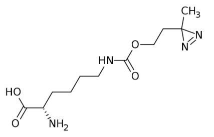	M. barkeri									M		A	F			8,9,10
		M. mazei								A				F			11,12
**10**	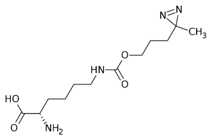	M. mazei								A				F			12
**11**	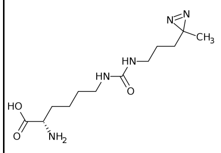	M. barkeri									A		S	F			13,14,15
**12**	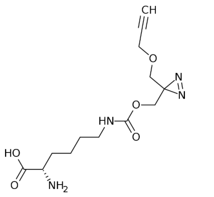	M. mazei								A				F			12
**13**	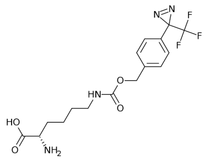	M. mazei								A				F			16
**14**	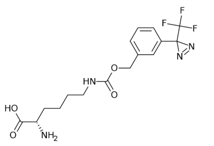	M. mazei	K		E					A				F			17
**15**	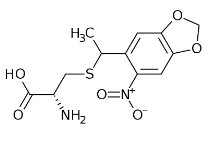	M. barkeri										Q	A		M		18,19

Table S1 List of reported PylRS mutants (continued)

Number	Structure	PylRS spieces	Mutation														Reference
		M. barkeri		D76		M241	L266	A267	L270	**Y271**	**L274**	N311	**C313**	**Y349**	V366	W382	
		M. mazei	R61		G131		L301	A302	L305	**Y306**	**L309**	N346	**C348**	**Y384**	V401	W417	
**16**	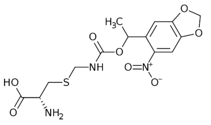	M. barkeri				F		S		C	M						20
**17**	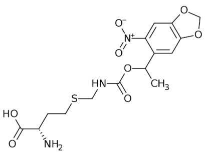	M. barkeri				F		S		C	M						20
**18**	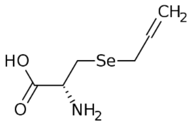	M. barkeri											W			T	21
**19**	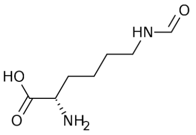	M. barkeri					M		I	F	A		F				22
**20**	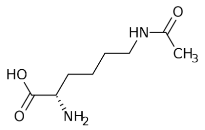	M. barkeri		G			V/M		I	F	A		F				23,24,25
		M. mazei					M		I	F/L	A		F				26,27,28
**21**	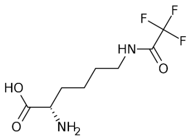	M. mazei					M			L	A		F				27
**22**	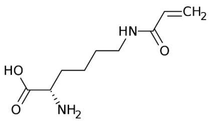	M. barkeri		G			M		I	F	A		F				29

Table S1 List of reported PylRS mutants (continued)

Number	Structure	PylRS spieces	Mutation														Reference
		M. barkeri		D76		M241	L266	A267	L270	**Y271**	**L274**	N311	**C313**	**Y349**	V366	W382	
		M. mazei	R61		G131		L301	A302	L305	**Y306**	**L309**	N346	**C348**	**Y384**	V401	W417	
**23**	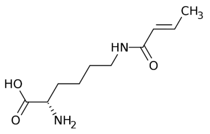	M. barkeri									A		F	F			8,30
**24**	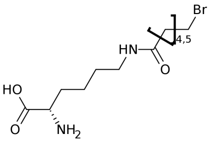	M. barkeri								M	A		A				31
**25**	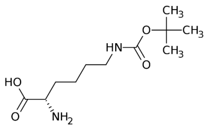	M. barkeri															26,32,33,34,<br>35,36,37
		M. mazei															8,18,23,25,<br>26,28,34,38,<br>39,40,41,42,<br>43,44,45,46
**26**	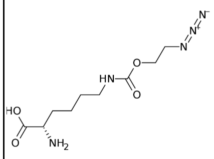	M. barkeri									A		S	F			8,13,32,33,<br>47,48,49,50,<br>51,52,53,54
		M. mazei												F			45
**27**	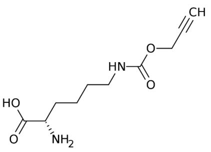	M. barkeri															8,40,55,56,<br>57
		M. mazei															31,40,49

Table S1 List of reported PylRS mutants (continued)

Number	Structure	PylRS spieces	Mutation														Reference
		M. barkeri		D76		M241	L266	A267	L270	**Y271**	**L274**	N311	**C313**	**Y349**	V366	W382	
		M. mazei	R61		G131		L301	A302	L305	**Y306**	**L309**	N346	**C348**	**Y384**	V401	W417	
**28**	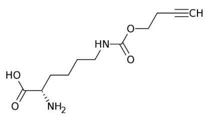	M. barkeri															33,37
		M. mazei															37
**29**	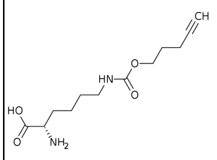	M. barkeri									A		S	F			14,49
**30**	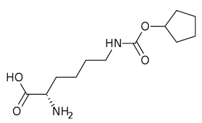	M. mazei															58
**31**	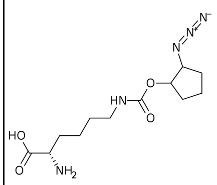	M. barkeri															14
**32**	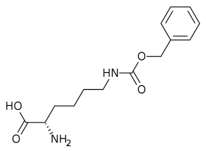	M. mazei	K		E					A	A		V	F			16,17,28
**33**	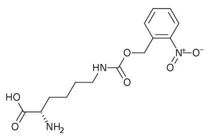	M. barkeri								I	A		A	F			14
		M. mazei								M	A		A	F			58,59,60

Table S1 List of reported PylRS mutants (continued)

**Number**	**Structure**	**PylRS spieces**	**Mutation**														**Reference**
		M. barkeri		D76		M241	L266	A267	L270	**Y271**	**L274**	N311	**C313**	**Y349**	V366	W382	
		M. mazei	R61		G131		L301	A302	L305	**Y306**	**L309**	N346	**C348**	**Y384**	V401	W417	
**34**	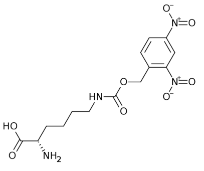	M. barkeri								M	T		A	F			61
**35**	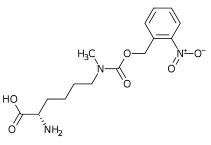	M. barkeri								I	M		A				62
**36**	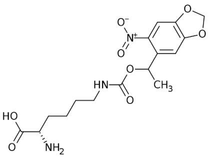	M. barkeri				F		S		C	M						20,35,40,63, 64,65,66,67, 68,69,70
**37**	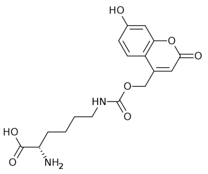	M. barkeri								A	M						71
**38**	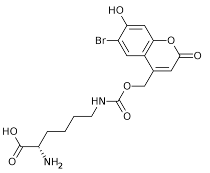	M. barkeri								A	M						71
**39**	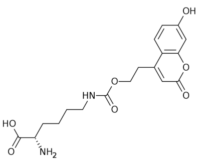	M. barkeri								A	M						71
**40**	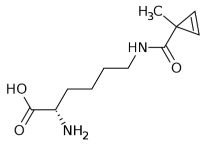	M. barkeri					M		I	L	A						72

Table S1 List of reported PylRS mutants (continued)

Number	Structure	PylRS spieces	Mutation														Reference
		M. barkeri		D76		M241	L266	A267	L270	**Y271**	**L274**	N311	**C313**	**Y349**	V366	W382	
		M. mazei	R61		G131		L301	A302	L305	**Y306**	**L309**	N346	**C348**	**Y384**	V401	W417	
**41**	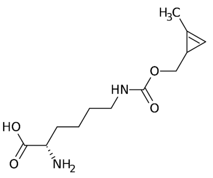	M. barkeri															33,47
		M. mazei								A				F			11,23,38,40, 42,73
**42**	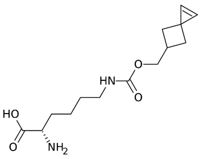	M. mazei															43
**43**	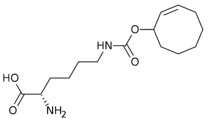	M. mazei								A				F			11,42,59,74, 76,76,77,78, 79,80,81,82, 83
**44**	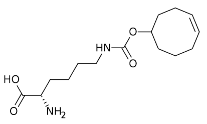	M. barkeri								A	M		A				42,84,85
		M. mazei								A	M		A	F			43,86
**45**	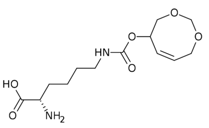	M. mazei								A				F			82

Table S1 List of reported PylRS mutants (continued)

Number	Structure	PylRS spieces	Mutation														Reference
		M. barkeri		D76		M241	L266	A267	L270	**Y271**	**L274**	N311	**C313**	**Y349**	V366	W382	
		M. mazei	R61		G131		L301	A302	L305	**Y306**	**L309**	N346	**C348**	**Y384**	V401	W417	
**46**	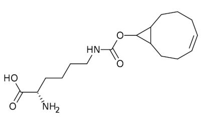	M. barkeri								A	M		A				84,85
		M. mazei								A				F			86
**47**	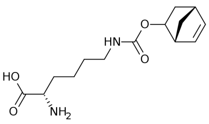	M. barkeri															47
		M. mazei								A				F			45,49,75,86
**48**	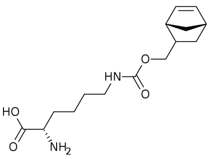	M. mazei								A				F			86
**49**	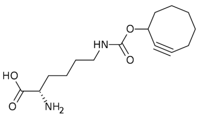	M. mazei								A				F			75,78,84,86, 87
**50**	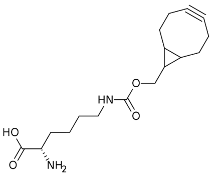	M. barkeri				F		S		M/C	G/M		A				14,85,88,89, 90,91
		M. mazei								A				F			11,12,42,46, 49,75,78,82, 83,84

[1] D. He, X. Xie, F. Yang, H. Zhang, H. Su, Y. Ge, H. Song, P. R. Chen, "Quantitative and Comparative Profiling of Protease Substrates through a Genetically Encoded Multifunctional Photocrosslinker" Angew. Chem. Int. Ed. 2017, 56, 14521.
[2] Y. Yang, H. Song, D. He, S. Zhang, S. Dai, X. Xie, S. Lin, Z. Hao, H. Zheng, P. R. Chen, "Genetically encoded releasable photo-cross-linking strategies for studying protein-protein interactions in living cells" Nat. Protoc. 2017, 12, 2147.
[3] Y. Tian, M. P. Jacinto, Y. Zeng, Z. Yu, J. Qu, W. R. Liu, Q. Lin, "Genetically Encoded 2-Aryl-5-carboxytetrazoles for Site-Selective Protein Photo-Cross-Linking" J. Am. Chem. Soc. 2017, 3, 6078.
[4] Y. Tian, Q. Lin, "Genetic encoding of 2-aryl-5-carboxytetrazole-based protein photo-cross-linkers" Chem. Commun. 2018, 54, 4449.
[5] A. Yamaguchi, T. Matsuda, K. Ohtake, T. Yanagisawa, S. Yokoyama, Y. Fujiwara, T. Watanabe, T. Hohsaka, K. Sakamot, "Incorporation of a Doubly Functionalized Synthetic Amino Acid into Proteins for Creating Chemical and Light-Induced Conjugates" Bioconjugate Chem. 2016, 27, 198.
[6] J. Luo, Q. Liu, K. Morihiro, A. Deiters, "Small-molecule control of protein function through Staudinger reductio" Nat. Chem. 2016, 8, 1027.
[7] Y. Ge, X. Fan, P. R. Chen, "A genetically encoded multifunctional unnatural amino acid for versatile protein manipulations in living cells" Chem. Sci. 2016, 7, 7055.
[8] A. Chatterjee, H. Xiao, M. Bollong, Hui-wang Ai, P. G. Schultz, "Efficient viral delivery system for unnatural amino acid mutagenesis in mammalian cells" Proc. Natl. Acad. Sci. U.S.A. 2013, 110, 11803.
[9] Hui-wang Ai, W. Shen, A. Sagi, P. R. Chen, P. G. Schultz, "Probing protein-protein interactions with a genetically encoded photo-crosslinking amino acid" ChemBioChem. 2011, 12, 1854.
[10] C. Chou, R. Uprety, L. Davis, J. W. Chin, A. Deiters, "Genetically encoding an aliphatic diazirine for protein photocrosslinking" Chem. Sci. 2011, 2, 480.
[11] B. Meineke, J. Heimgartner, L. Lafranchi, S. J. Elsässer, "Methanomethylophilus alvus Mx1201 provides basis for mutual orthogonal pyrrolysyl tRNA/Aminoacyl-tRNA synthetase pairs in mammalian cells" ACS Chem. Biol. 2018, 13, 3087.
[12] J. E. Hoffmann, D. Dziuba, F. Stein, C. Schultz, "A bifunctional noncanonical amino acid: synthesis, expression, and residue-specific proteome-wide incorporation" Biochemistry 2018, 57, 4747.
[13] Y. Zheng, F. Yu, Y. Wu, L. Si, H. Xu, C. Zhang, Q. Xia, S. Xiao, Q. Wang, Q. He, P. Chen, J. Wang, K. Taira, L. Zhang, D. Zhou, "Broadening the versatility of lentiviral vectors as a tool in nucleic acid research via genetic code expansion" Nucleic Acids Res. 2015, 43, e73.
[14] S. Lin, H. Yan, L. Li, M. Yang, B. Peng, S. Chen, W. Li, P. R. Chen, "Site-specific engineering of chemical functionalities on the surface of live hepatitis D virus" Angew. Chem. Int. Ed. 2013, 52, 13970.
[15] C. Zhang, J. Lu, H. Su, J. Yang, D. Zhou, "Fatty acid synthase cooperates with protrudin to facilitate membrane outgrowth of cellular protrusions" Sci. Rep. 2017, 7, 46569.
[16] T. Yanagisawa, N. Hino, F. Iraha, T. Mukai, K. Sakamoto, S. Yokoyama, "Wide-range protein photo-crosslinking achieved by a genetically encoded Nε-(benzyloxycarbonyl)lysine derivative with a diazirinyl moiety" Mol. Biosyst. 2012, 8, 1131.
[17] A. Kita, N. Hino, S. Higashi, K. Hirota, R. Narumi, J. Adachi, K. Takafuji, K. Ishimoto, Y. Okada, K. Sakamoto, T. Tomonaga, S. Takashima, H. Mizuguchi, T. Doi, "Adenovirus vector-based incorporation of a photo-cross-linkable amino acid into proteins in human primary cells and cancerous cell lines" Sci. Rep. 2016, 6, 36946.
[18] D. P. Nguyen, M. Mahesh, S. J. Elsässer, S. M. Hancock, C. Uttamapinant, J. W. Chin, "Genetic encoding of photocaged cysteine allows photoactivation of TEV protease in live mammalian cells" J. Am. Chem. Soc. 2014, 136, 2240.
[19] J. A. Gramespacher, A. J. Stevens, D. P. Nguyen, J. W. Chin, T. W. Muir, "Intein zymogens: conditional assembly and splicing of split inteins via targeted proteolysis" J. Am. Chem. Soc. 2017, 139, 8074.
[20] R. Uprety, J. Luo, J. Liu, Y. Naro, S. Samanta, A. Deiters, "Genetic encoding of caged cysteine and caged homocysteine in bacterial and mammalian cells" ChemBioChem 2014, 15, 1793.
[21] J. Liu, F. Zheng, R. Cheng, S. Li, S. Rozovsky, Q. Wang, L. Wang, "Site-specific incorporation of selenocysteine using an expanded genetic code and palladium-mediated chemical deprotection" J. Am. Chem. Soc. 2018, 140, 8807.
[22] T. Wang, Q. Zhou, F. Li, Y. Yu, X. Yin, J. Wang, "Genetic incorporation of N(epsilon)-Formyllysine, a new histone post-translational modification" ChemBioChem 2015, 16, 1440.
[23] S. J. Elsässer, R. J. Ernst, O. S. Walker, J. W. Chin, "Genetic code expansion in stable cell lines enables encoded chromatin modification" Nat. Methods 2016, 13, 158.
[24] W. Xuan, A. Yao, P. G. Schultz, "Genetically encoded fluorescent probe for detecting sirtuins in living cells" J. Am. Chem. Soc. 2017, 139, 12350.
[25] S. Cohen, E. Arbely, "Single-plasmid-based system for efficient noncanonical amino acid mutagenesis in cultured mammalian cells" ChemBioChem 2016, 17, 1008.
[26] P. Mali, D. Katrekar, "In vivo RNA targeting of point mutations via suppressor tRNAs and adenosine deaminases" bioRxiv ,
[27] S. Han, A. Yang, S. Lee, H. W. Lee, C. B. Park, H. S. Park, "Expanding the genetic code of Mus musculus" Nat. Commun. 2017, 8, 14568.
[28] T. Mukai, T. Kobayashi, N. Hino, T. Yanagisawa, K. Sakamoto, S. Yokoyama, "Adding l-lysine derivatives to the genetic code of mammalian cells with engineered pyrrolysyl-tRNA synthetases" Biochem. Biophys. Res. Commun. 2008, 371, 818.

[29] F. Li, H. Zhang, Y. Sun, Y. Pan, J. Zhou, J. Wang, "Expanding the genetic code for photoclick chemistry in E. coli, mammalian cells, and A. thaliana" Angew. Chem. Int. Ed. 2013, 52, 9700.
[30] C. H. Kim, M. Kang, H. J. Kim, A. Chatterjee, P. G. Schultz, "Site-specific incorporation of epsilon-N-crotonyllysine into histones" Angew. Chem. Int. Ed. 2012, 51, 7246.
[31] M. Cigler, T. G. Müller, D. Horn-Ghetko, Marie-Kristin von Wrisberg, M. Fottner, R. S. Goody, A. Itzen, M. P. Müller, K. Lang, "Proximity-triggered covalent stabilization of low-affinity protein complexes in vitro and in vivo" Angew. Chem. Int. Ed. 2017, 56, 15737.
[32] H. Xiao, A. Chatterjee, S. H. Choi, K. M. Bajjuri, S. C. Sinha, P. G. Schultz, "Genetic incorporation of multiple unnatural amino acids into proteins in mammalian cells" Angew. Chem. Int. Ed. 2013, 52, 14080.
[33] Y. Zheng, P. S. Addy, R. Mukherjee, A. Chatterjee, "Defining the current scope and limitations of dual noncanonical amino acid mutagenesis in mammalian cells" Chem. Sci. 2017, 8, 7211.
[34] V. Beranek, J. C. W. Willis, J. W. Chin, "An evolved Methanomethylophilus alvus pyrrolysyl-tRNA Synthetase/tRNA pair is highly active and orthogonal in mammalian cells" Biochemistry 2019, 58, 387.
[35] A. Gautier, D. P. Nguyen, H. Lusic, W. An, A. Deiters, J. W. Chin, "Genetically encoded photocontrol of protein localization in mammalian cells" J. Am. Chem. Soc. 2010, 132, 4086.
[36] N. Schneider, C. Gäbelein, J. Wiener, T. Georgiev, N. Gobet, W. Weber, M. Meier, "Genetic code expansion method for temporal labeling of endogenously expressed proteins" ACS Chem. Biol. 2018, 13, 3049.
[37] N. Li, C. P. Ramil, R. K. Lim, Q. Lin, "A genetically encoded alkyne directs palladium-mediated protein labeling on live mammalian cell surface" ACS Chem. Biol. 2015, 10, 379.
[38] W. H. Schmied, S. J. Elsässer, C. Uttamapinant, J. W. Chin, "Efficient multisite unnatural amino acid incorporation in mammalian cells via optimized pyrrolysyl tRNA synthetase/tRNA expression and engineered eRF1" J. Am. Chem. Soc. 2014, 136, 15577.
[39] T. Suzuki, M. Asami, S. G. Patel, L. Y. P. Luk, Yu-Hsuan Tsai, A. C. F. Perry, "Switchable genome editing via genetic code expansion" Sci. Rep. 2018, 8, 10051.
[40] R. J. Ernst, T. P. Krogager, E. S. Maywood, R. Zanchi, V. Beránek, T. S. Elliott, N. P. Barry, M. H. Hastings, J. W. Chin, "Genetic code expansion in the mouse brain" Nat. Chem. Biol. 2016, 12, 776.
[41] V. K. Lacey, G. V. Louie, J. P. Noel, L. Wang, "Expanding the library and substrate diversity of the pyrrolysyl-tRNA synthetase to incorporate unnatural amino acids containing conjugated rings" ChemBioChem 2013, 14, 2100.
[42] T. Peng, H. C. Hang, "Site-specific bioorthogonal labeling for fluorescence imaging of intracellular proteins in living cells" J. Am. Chem. Soc. 2016, 138, 14423.

[43] C. P. Ramil, M. Dong, P. An, T. M. Lewandowski, Z. Yu, L. J. Miller, Q. Lin, "Spirohexene-tetrazine ligation enables bioorthogonal labeling of class B G protein-coupled receptors in live cells" J. Am. Chem. Soc. 2017, 139, 13376.

[44] E. Arbely, J. Torres-Kolbus, A. Deiters, J. W. Chin, "Photocontrol of tyrosine phosphorylation in mammalian cells via genetic encoding of photocaged tyrosine" J. Am. Chem. Soc. 2012, 134, 11912.
[45] K. Lang, L. Davis, J. Torres-Kolbus, C. Chou, A. Deiters, J. W. Chin, "Genetically encoded norbornene directs site-specific cellular protein labelling via a rapid bioorthogonal reaction" Nat. Chem. 2012, 4, 298.
[46] N. Aloush, T. Schvartz, A. I. König, S. Cohen, E. Brozgol, B. Tam, D. Nachmias, O. Ben-David, Y. Garini, N. Elia, E. Arbely, "Live cell imaging of bioorthogonally labelled proteins generated with a single pyrrolysine tRNA gene" Sci. Rep. 2018, 8, 14527.
[47] Y. Zheng, R. Mukherjee, M. A. Chin, P. Igo, M. J. Gilgenast, A. Chatterjee, "Expanding the scope of single- and double-noncanonical amino acid mutagenesis in mammalian cells using orthogonal polyspecific leucyl-tRNA synthetases" Biochemistry 2018, 57, 441.
[48] L. Si, H. Xu, X. Zhou, Z. Zhang, Z. Tian, Y. Wang, Y. Wu, B. Zhang, Z. Niu, C. Zhang, G. Fu, S. Xiao, Q. Xia, L. Zhang, D. Zhou, "Generation of influenza A viruses as live but replication-incompetent virus vaccines" Science 2016, 354, 1170.
[49] Y. Yang, S. Lin, W. Lin, P. R. Chen, "Ligand-assisted dual-site click labeling of EGFR on living cells" ChemBioChem 2014, 15, 1738.
[50] C. Zhang, T. Yao, Y. Zheng, Z. Li, Q. Zhang, L. Zhang, D. Zhou, "Development of next generation adeno-associated viral vectors capable of selective tropism and efficient gene delivery" Biomaterials 2016, 80, 134.
[51] Z. Zhang, H. Xu, L. Si, Y. Chen, B. Zhang, Y. Wang, Y. Wu, X. Zhou, L. Zhang, D. Zhou, "Construction of an inducible stable cell line for efficient incorporation of unnatural amino acids in mammalian cells" Biochem. Biophys. Res. Commun. 2017, 489, 490.
[52] T. Yao, X. Zhou, C. Zhang, X. Yu, Z. Tian, L. Zhang, D. Zhou, "Site-specific PEGylated adeno-associated viruses with increased serum stability and reduced immunogenicity" Molecules 2017, 22, 1155.
[53] D. Katrekar, A. M. Moreno, G. Chen, A. Worlikar, P. Mali, "Oligonucleotide conjugated multi-functional adeno-associated viruses" Sci. Rep. 2018, 8, 3589.
[54] R. E. Kelemen, R. Mukherjee, X. Cao, S. B. Erickson, Y. Zheng, A. Chatterjee, "A precise chemical strategy to alter the receptor specificity of the adeno-associated virus" Angew. Chem. Int. Ed. 2016, 55, 10645.
[55] J. Li, J. Yu, J. Zhao, J. Wang, S. Zheng, S. Lin, L. Chen, M. Yang, S. Jia, X. Zhang, P. R. Chen, "Palladium-triggered deprotection chemistry for protein activation in living cells" Nat. Chem. 2014, 6, 352.
[56] J. Zhang, S. Yan, Z. He, C. Ding, T. Zhai, Y. Chen, H. Li, G. Yang, X. Zhou, P. Wang, "Small unnatural amino acid carried raman tag for molecular imaging of genetically targeted proteins" J. Phys. Chem. Lett. 2018, 9, 4679.

[57] T. Lühmann, G. Jones, M. Gutmann, Jens-Christoph Rybak, J. Nickel, M. Rubini, L. Meinel, "Bio-orthogonal immobilization of fibroblast growth factor 2 for spatial controlled cell proliferation" ACS Biomater. Sci. Eng. 2015, 1, 740.

[58] P. R. Chen, D. Groff, J. Guo, W. Ou, S. Cellitti, B. H. Geierstanger, P. G. Schultz, "A facile system for encoding unnatural amino acids in mammalian cells" Angew. Chem. Int. Ed. 2009, 48, 4052.

[59] X. Fan, Y. Ge, F. Lin, Y. Yang, G. Zhang, W. S. C. Ngai, Z. Lin, S. Zheng, J. Wang, J. Zhao, J. Li, P. R. Chen, "Optimized tetrazine derivatives for rapid bioorthogonal decaging in living cells" Angew. Chem. Int. Ed. 2016, 55, 14046.

[60] J. Zhao, S. Lin, Y. Huang, J. Zhao, P. R. Chen, "Mechanism-based design of a photoactivatable firefly luciferase" J. Am. Chem. Soc. 2013, 135, 7410.

[61] W. Ren, A. Ji, M. X. Wang, Hui-wang Ai, "Expanding the Genetic Code for a Dinitrophenyl Hapten" ChemBioChem 2015, 16, 2007.

[62] D. Groff, P. R. Chen, F. B. Peters, P. G. Schultz, "A genetically encoded epsilon-N-methyl lysine in mammalian cells" ChemBioChem 2010, 11, 1066.

[63] A. Liaunardy-Jopeace, B. L. Murton, M. Mahesh, J. W. Chin, J. R. James, "Encoding optical control in LCK kinase to quantitatively investigate its activity in live cells" Nat. Struct. Mol. Biol. 2017, 24, 1155.

[64] O. S. Walker, S. J. Elsässer, M. Mahesh, M. Bachman, S. Balasubramanian, J. W. Chin, "Photoactivation of mutant isocitrate dehydrogenase 2 reveals rapid cancer-associated metabolic and epigenetic changes" J. Am. Chem. Soc. 2016, 138, 718.

[65] S. B. Erickson, R. Mukherjee, R. E. Kelemen, C. J. J. Wrobel, X. Cao, A. Chatterjee, "Precise photoremovable perturbation of a virus–host interaction" Angew. Chem. Int. Ed. 2017, 56, 4234.

[66] J. Luo, E. Arbely, J. Zhang, C. Chou, R. Uprety, J. W. Chin, A. Deiters, "Genetically encoded optical activation of DNA recombination in human cells" Chem. Commun. 2016, 52, 8529.

[67] H. Engelke, C. Chou, R. Uprety, P. Jess, A. Deiters, "Control of protein function through optochemical translocation" ACS Synth. Biol. 2014, 3, 731.

[68] J. Hemphill, E. K. Borchardt, K. Brown, A. Asokan, A. Deiters, "Optical control of CRISPR/Cas9 gene editing" J. Am. Chem. Soc. 2015, 137, 5642.

[69] A. Gautier, A. Deiters, J. W. Chin, "Light-activated kinases enable temporal dissection of signaling networks in living cells" J. Am. Chem. Soc. 2011, 133, 2124.

[70] J. Hemphill, C. Chou, J. W. Chin, A. Deiters, "Genetically encoded light-activated transcription for spatiotemporal control of gene expression and gene silencing in mammalian cells" J. Am. Chem. Soc. 2013, 135, 13433.

[71] J. Luo, R. Uprety, Y. Naro, C. Chou, D. P. Nguyen, J. W. Chin, A. Deiters, "Genetically encoded optochemical probes for simultaneous fluorescence reporting and light activation of protein function with two-photon excitation" J. Am. Chem. Soc. 2014, 136, 15551.

[72] Z. Yu, Y. Pan, Z. Wang, J. Wang, Q. Lin, "Genetically encoded cyclopropene directs rapid, photoclick-chemistry-mediated protein labeling in mammalian cells" Angew. Chem. Int. Ed. 2012, 51, 10600.

[73] T. S. Elliott, F. M. Townsley, A. Bianco, R. J. Ernst, A. Sachdeva, S. J. Elsässer, L. Davis, K. Lang, R. Pisa, S. Greiss, K. S. Lilley, J. W. Chin, "Proteome labeling and protein identification in specific tissues and at specific developmental stages in an animal" Nat. Biotechnol. 2014, 32, 465.

[74] S. Zheng, X. Fan, J. Wang, J. Zhao, P. R. Chen, "Dissection of kinase isoforms through orthogonal and chemical inducible signaling cascades" ChemBioChem 2017, 18, 1593.

[75] I. Nikić, J. H. Kang, G. E. Girona, I. V. Aramburu, E. A. Lemke, "Labeling proteins on live mammalian cells using click chemistry" Nat. Protoc. 2015, 10, 780.

[76] J. Li, S. Jia, P. R. Chen, "Diels-Alder reaction-triggered bioorthogonal protein decaging in living cells" Nat. Chem. Biol. 2014, 10, 1003.

[77] G. Zhang, J. Li, R. Xie, X. Fan, Y. Liu, S. Zheng, Y. Ge, P. R. Chen, "Bioorthogonal chemical activation of kinases in living systems" ACS Cent. Sci. 2016, 2, 325.

[78] I. Nikić, T. Plass, O. Schraidt, J. Szymański, J. A. G. Briggs, C. Schultz, E. A. Lemke, "Minimal tags for rapid dual-color live-cell labeling and super-resolution microscopy" Angew. Chem. Int. Ed. 2014, 53, 2245.

[79] I. Nikić, G. E. Girona, J. H. Kang, G. Paci, S. Mikhaleva, C. Koehler, N. V. Shymanska, C. V. Santos, D. Spitz, E. A. Lemke, "Debugging eukaryotic genetic code expansion for site-specific Click-PAINT super-resolution microscopy" Angew. Chem. Int. Ed. 2016, 55, 16172.

[80] A. Rutkowska, T. Plass, J. E. Hoffmann, D. A. Yushchenko, S. Feng, C. Schultz, "T-CrAsH: a heterologous chemical crosslinker" ChemBioChem 2014, 15, 1765.

[81] Jan-Erik Hoffmann, T. Plass, I. Nikić, I. V. Aramburu, C. Koehler, H. Gillandt, E. A. Lemke, C. Schultz, "Highly stable trans-cyclooctene amino acids for live-cell labeling" Chem. Eur. J. 2015, 21, 12266.

[82] E. Kozma, I. Nikić, B. R. Varga, I. V. Aramburu, J. H. Kang, O. T. Fackler, E. A. Lemke, P. Kele, "Hydrophilic trans-cyclooctenylated noncanonical amino acids for fast intracellular protein labeling" ChemBioChem 2016, 17, 1518.

[83] V. Sakin, J. Hanne, J. Dunder, M. Anders-Össwein, V. Laketa, I. Nikić, Hans-Georg Kräusslich, E. A. Lemke, B. Mü ller, "A versatile tool for live-cell imaging and super-resolution nanoscopy studies of hiv-1 env distribution and mobility" Cell Chem. Biol. 2017, 24, 635.

[84] K. Lang, L. Davis, S. Wallace, M. Mahesh, D. J. Cox, M. L. Blackman, J. M. Fox, J. W. Chin, "Genetic encoding of bicyclononynes and trans-cyclooctenes for site-specific protein labeling in vitro and in live mammalian cells via rapid fluorogenic Diels-Alder reactions" J. Am. Chem. Soc. 2012, 134, 10317.

[85] C. Uttamapinant, J. D. Howe, K. Lang, V. Beránek, L. Davis, M. Mahesh, N. P. Barry, J. W. Chin, "Genetic code expansion enables live-cell and super-resolution imaging of site-specifically labeled cellular proteins" J. Am. Chem. Soc. 2015, 137, 4602.

[86] T. Plass, S. Milles, C. Koehler, J. Szymański, R. Mueller, M. Wiessler, C. Schultz, E. A. Lemke, "Amino acids for Diels-Alder reactions in living cells" Angew. Chem. Int. Ed. 2012, 51, 4166.
[87] L. Xue, E. Prifti, K. Johnsson, "A general strategy for the semisynthesis of ratiometric fluorescent sensor proteins with increased dynamic range" J. Am. Chem. Soc. 2016, 138, 5258.

[88] M. Baumdick, M. Gelleri, C. Uttamapinant, V. Beránek, J. W. Chin, P. I. H. Bastiaens, "A conformational sensor based on genetic code expansion reveals an autocatalytic component in EGFR activation" Nat. Commun. 2018, 9, 3847.

[89] Yu-Hsuan Tsai, S. Essig, J. R. James, K. Lang, J. W. Chin, "Selective, rapid and optically switchable regulation of protein function in live mammalian cells" Nat. Chem. 2015, 7, 554.
[90] A. Borrmann, S. Milles, T. Plass, J. Dommerholt, J. M. M. Verkade, M. Wiessler, C. Schultz, J. C. M. van Hest, F. L. van Delft, E. A. Lemke, "Genetic encoding of a bicyclo[6.1.0]nonyne-charged amino acid enables fast cellular protein imaging by metal-free ligation" ChemBioChem 2012, 13, 2094.
[91] T. Schvartz, N. Aloush, I. Goliand, I. Segal, D. Nachmias, E. Arbely, N. Elia, "Direct fluorescent-dye labeling of alpha-tubulin in mammalian cells for live cell and superresolution imaging" Mol. Biol. Cell. 2017, 28, 2747.
