# Supplementary material for: Genetic Encoding of a Trifunctional Photo‐Cross‐Linker with a Cleavable Alkyl Ester Moiety
Source: Chembiochem. 2026 Jan 30;27(3):e202500827. doi: 10.1002/cbic.202500827 (PMC12859177; doi:10.1002/cbic.202500827)
Supplement: Supplementary file 1 — Supplementary Material [file CBIC-27-e202500827-s001.zip › MT_Revision Supporting Information submit.pdf]

Supporting Information

Genetic Encoding of a Trifunctional Photo-Cross-Linker with a Cleavable Alkyl Ester Moiety

Masahiro Takayama+[a,b], Tomoya Tsubota+[a], Takao Yamaguchi[a], Kosuke Chiba[a], Takumi Yoshida[a], Yoshiyuki Hari[c], Yu-Shi Tian[a], Daisuke Takaya[a], Asuka Mori[a], Tomohito Tsukamoto[a], Kenji Ishimoto[a], Yukio Ago[a,d], Yoshiaki Okada[a,g], Kensaku Sakamoto[e,f], Takefumi Doi[a], Kaori Fukuzawa[a], Satoshi Obika[a], Shinsaku Nakagawa[a], Nobumasa Hino*[a,g]

[a] M Takayama, T Tsubota, T Yamaguchi, K Chiba, T Yoshida, YS Tian, D Takaya, A Mori, T Tsukamoto, K Ishimoto, Y Okada, T Doi, K Fukuzawa, S Obika, S Nakagawa, N Hino.
Graduate School of Pharmaceutical Sciences,
The University of Osaka, 1-6 Yamadaoka, Suita, Osaka 565-0871, Japan.
E-mail: hino@phs.osaka-u.ac.jp
[b] M Takayama.
Shionogi Pharmaceutical Research Center,
Shionogi & Co., Ltd.,
3-1-1, Futaba-cho, Toyonaka, Osaka, 561-0825, Japan.
[c] Y Hari.
Faculty of Pharmaceutical Sciences,
Tokushima Bunri University,
Nishihama, Yamashiro-cho, Tokushima, Tokushima 770-8514, Japan.
[d] Y Ago.
Department of Cellular and Molecular Pharmacology, Graduate School of Biomedical and Health Sciences,
Hiroshima University,
1-2-3 Kasumi, Minami-ku, Hiroshima, Hiroshima, 734-8553, Japan.
[e] K Sakamoto.
Laboratory for Nonnatural Amino Acid Technology, RIKEN Center for Biosystems Dynamics Research, RIKEN
1-7-22 Suehiro-cho, Tsurumi-ku, Yokohama, Kanagawa 230-0045, Japan.
[f] K Sakamoto.
Department of Drug Target Protein Research, Shinshu University School of Medicine,
3-1-1 Asahi, Matsumoto, Nagano, 390-8621, Japan.
[g] Y Okada, N Hino.
Center for Infectious Disease Education and Research,
The University of Osaka,
1-10 Yamadaoka, Suita, Osaka 565-0871, Japan.

List of contents

Experimental Section	S-3
Figure S1	S-4 ~ S-5
Figure S2	S-6
Figure S3	S-7
Table S2	S-8 ~ S-9
Synthesis of novel PAAs	S-10 ~ S-20
Scheme S1	S-10 ~ S-13
Scheme S2	S-14 ~ S-17
Scheme S3	S-18 ~ S-20
References	S-21

Experimental Section

nLC/MS/MS analysis of ncAA-containing peptides

The proteins were purified by sp3 method as described [1,2]. The sp3 bead mix was added to the samples at a ratio of 1:8 (protein to brads). For reduction, 10 mM DTT was added to the samples and incubated at 95 °C for 5 min. For alkylation, 30 mM 2-iodoacetamide was added to the reduced samples and incubated at 25 °C for 30 min under light-shielded condition. The proteins were digested on sp3 beads by trypsin at a ratio of 1:50 (enzyme to protein) in 100 mM Tris-HCl (pH 7.5) at 37 °C for 18 h. After digestion, the solutions were acidified with 1% trifluoroacetic acid (TFA), and the resulting supernatant was collected in a new tube. These samples were injected on a Hypersil Gold column (Thermo Scientific [Waltham, MA, USA]) and a 3 μm C18 nano HPLC capillary column (Nikkyo Technos [Tokyo, Japan]) using a Vanquish Neo UHPLC system (Thermo Scientific). Peptides were separated and eluted from the column with buffer B (100% acetonitrile, 0.1% formic acid) at a flow rate of 300 nL/min with a gradient of 2-40%, 60 min. All samples were analyzed on Orbitrap Exploris 240 (Thermo Scientific) mass spectrometer coupled to Vanquish Neo system. The mass spectrometer was operated in positive mode with the following settings: spray voltage, 2000 V; IT tube temperature, 275 °C; HCD collision energy, 30%; resolution, 60000; RFLens, 70%; Intensity threshold, 5.0e3; charge, 2-9; and scan range, 200-2000.

Figure S1. Small-scale screening of *M. mazei* PylRS mutants that incorporate newly designed PAAs more efficiently

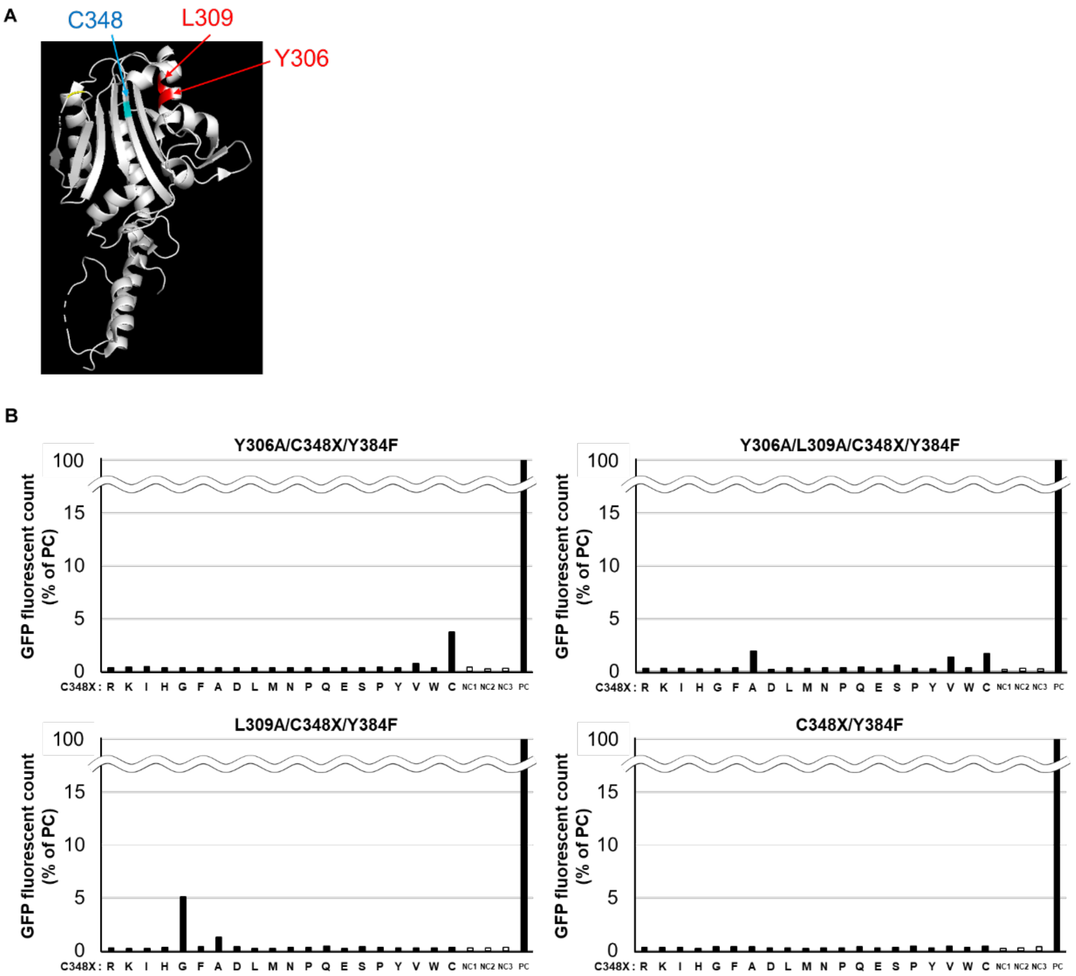

C

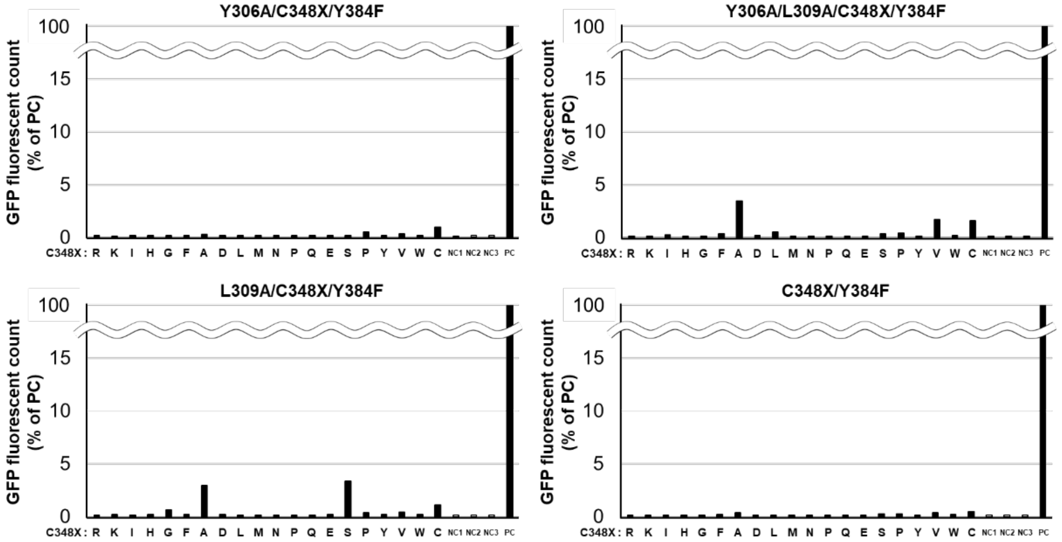

A) 3D structure of the *Mm*PylRS (gray, PDB ID: 2E3C). The residues Y306 and L309, which are substituted with alanine, are shown in red; the C348 residue, which is substituted with an arbitrary amino acid, is shown in cyan. B) Screening of *Mm*PylRS mutants that recognize mAAAsu using EGFP(33TAG) as a reporter. Cells (293 c18) were transfected with plasmids encoding each *Mm*PylRS mutant, tRNA^{Pyl}, and EGFP(33TAG) and incubated in the presence or absence of 100 µM of mAAAsu for 24 h. The fluorescence intensities of the lysates were measured as described in Figure 2. The fluorescent counts are shown relative to the positive control (PC) expressing wild-type EGFP, set at 100%. The wavy line indicates a break in the y-axis. Negative control 1 (NC1) indicates cells cultured in the absence of mAAAsu. Negative control 2 (NC2) and 3 (NC3) indicate cells transfected without tRNA^{Pyl} and *Mm*PylRS, respectively. C) Screening of the *Mm*PylRS mutants that recognize DiZAAsu. The analysis was similarly performed as described in (B), using DiZAAsu instead of mAAAsu.

Figure S2. Normalized comparison of PAA-incorporation efficiencies between conventional and newly screened *Mm*PylRS mutants

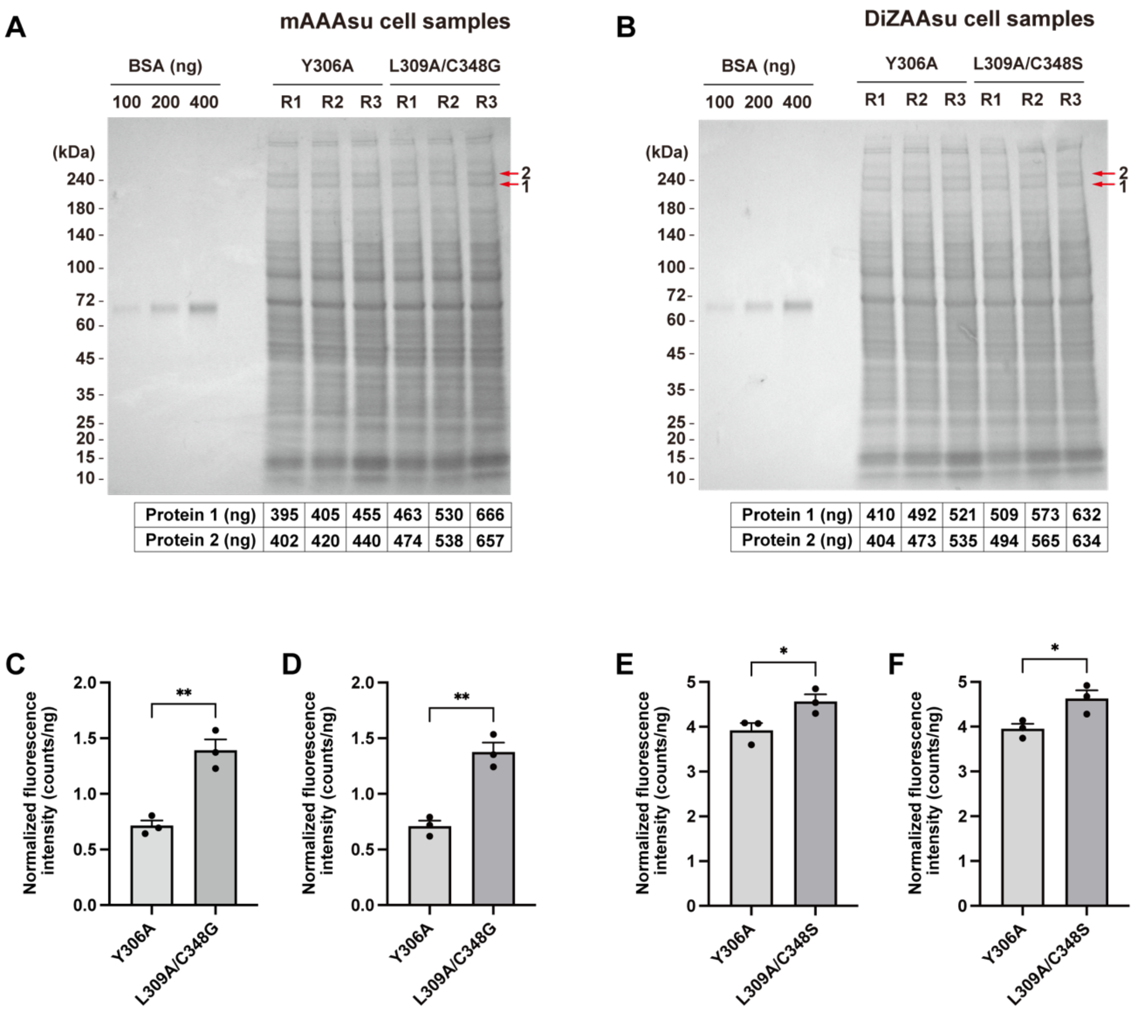

Protein 1 (ng)	395	405	455	463	530	666
Protein 2 (ng)	402	420	440	474	538	657

Protein 1 (ng)	410	492	521	509	573	632
Protein 2 (ng)	404	473	535	494	565	634

A, B) Coomassie Brilliant Blue staining of the cell lysates used for the fluorescence measurements in Figure 2D (for mAAAsu) and Figure 2E (for DiZAAsu). R1, R2, and R3 represent replicates 1, 2, and 3, respectively. The protein amounts of two prominent protein bands, indicated as Protein 1 and Protein 2, were quantified using a BSA standard curve and used for normalization. C, D) Comparison of mAAAsu incorporation efficiencies calculated by normalizing the fluorescence intensity counts (from Figure 2D) to the amount of Protein 1 (C) and Protein 2 (D). E, F) Comparison of DiZAAsu incorporation efficiencies calculated by normalizing the fluorescence intensity counts (from Figure 2E) to the amount of Protein 1 (E) and Protein 2 (F). In all cases, the optimized mutants showed significantly higher efficiency. The values represent mean ± SE (n = 3). *$p < 0.05$, **$p < 0.01$ (calculated using an unpaired Student's t-test).

Figure S3. Site-specific incorporation of Asu-based ncAAs at position 33 of EGFP

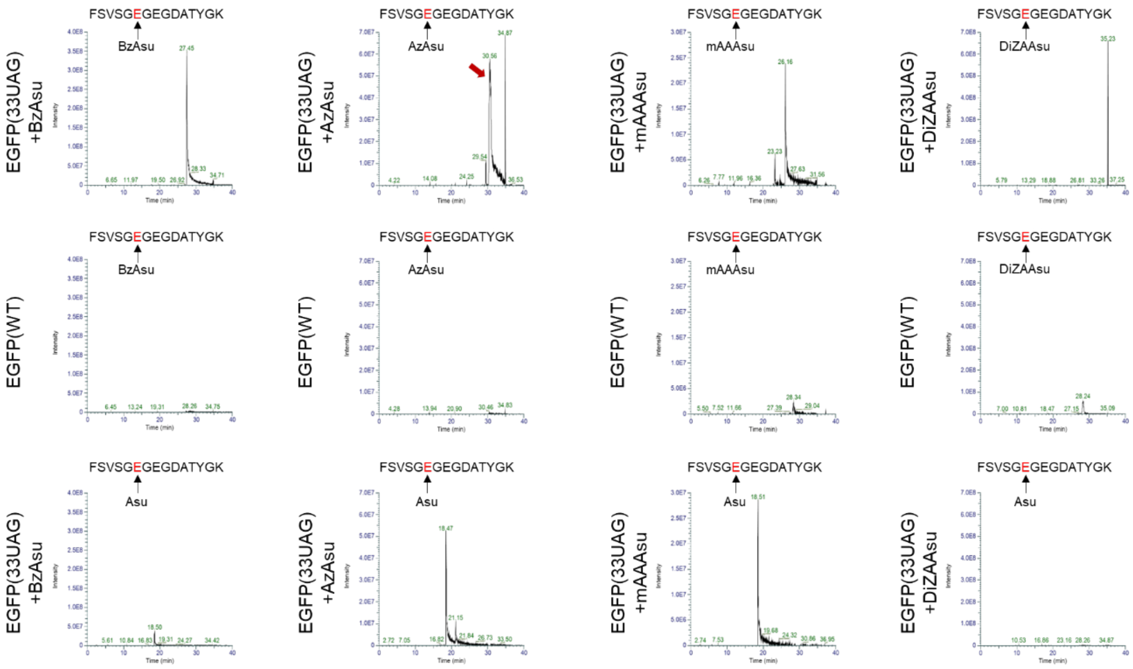

Asu-based ncAAs-incorporated EGFP-FLAG or wild-type EGFP-FLAG were expressed in 293 c18 cells. The proteins were purified via their FLAG tags, digested and further purified by sp3 method. After reduction and alkylation, the proteins were digested on sp3 beads by trypsin, analyzed by nLC/MS/MS. The extracted ion chromatograms (XICs) of trypsin-digested peptides containing the ncAA-incorporated residue at position 33, derived from the ncAA-incorporated EGFP, are shown in the upper panels. The equivalent XICs in the MS1 data from wild-type EGFP are shown in the middle panels. In all cases, almost no peaks are detected. The XICs of the trypsin-digested peptides containing Asu-incorporated residue 33 derived from the ncAA-incorporated EGFP are shown in the lower panels. Mass tolerance was set at 10 parts per million (ppm).

Table S2. List of DNA primers for site-directed mutagenesis

DNA primers		
PylRS_C348A	forward	5'-CCATGCTGAACTTCGCCCAGATGGGATCG-3'
	reverse	5'-CGATCCCATCTGGGCGAAGTTCAGCATGG-3'
PylRS_C348D	forward	5'-CCATGCTGAACTTCGACCAGATGGGATCG-3'
	reverse	5'-CGATCCCATCTGGTCGAAGTTCAGCATGG-3'
PylRS_C348E	forward	5'-CCATGCTGAACTTCGAGCAGATGGGATCG-3'
	reverse	5'-CGATCCCATCTGCTCGAAGTTCAGCATGG-3'
PylRS_C348F	forward	5'-CATGCTGAACTTCTTCCAGATGGGATC-3'
	reverse	5'-GATCCCATCTGGAAGAAGTTCAGCATG-3'
PylRS_C348G	forward	5'-CATGCTGAACTTCGGCCAGATGGG-3'
	reverse	5'-CCCATCTGGCCGAAGTTCAGCATG-3'
PylRS_C348H	forward	5'-CCATGCTGAACTTCCACCAGATGGGATCG-3'
	reverse	5'-CGATCCCATCTGGTGGAAGTTCAGCATGG-3'
PylRS_C348I	forward	5'-CCATGCTGAACTTCATCCAGATGGGATCG-3'
	reverse	5'-CGATCCCATCTGGATGAAGTTCAGCATGG-3'
PylRS_C348K	forward	5'-CCATGCTGAACTTCAAGCAGATGGGATCG-3'
	reverse	5'-CGATCCCATCTGCTTGAAGTTCAGCATGG-3'
PylRS_C348L	forward	5'-CATGCTGAACTTCTTGCAGATGGGATCG-3'
	reverse	5'-CGATCCCATCTGCAAGAAGTTCAGCATG-3'
PylRS_C348M	forward	5'-CCATGCTGAACTTCATGCAGATGGGATCG-3'
	reverse	5'-CGATCCCATCTGCATGAAGTTCAGCATGG-3'
PylRS_C348N	forward	5'-CCATGCTGAACTTCAACCAGATGGGATCG-3'
	reverse	5'-CGATCCCATCTGGTTGAAGTTCAGCATGG-3'
PylRS_C348P	forward	5'-CCATGCTGAACTTCCCCCAGATGGGATCG-3'
	reverse	5'-CGATCCCATCTGGGGGAAGTTCAGCATGG-3'
PylRS_C348Q	forward	5'-CCATGCTGAACTTCCAGCAGATGGGATCG-3'
	reverse	5'-CGATCCCATCTGCTGGAAGTTCAGCATGG-3'
PylRS_C348R	forward	5'-CATGCTGAACTTCCGCCAGATGGG-3'
	reverse	5'-CCCATCTGGCGGAAGTTCAGCATG-3'
PylRS_C348S	forward	5'-CATGCTGAACTTCAGCCAGATGGG-3'
	reverse	5'-CCCATCTGGCTGAAGTTCAGCATG-3'
PylRS_C348T	forward	5'-CCATGCTGAACTTCACCCAGATGGGATCG-3'
	reverse	5'-CGATCCCATCTGGGTGAAGTTCAGCATGG-3'

PylRS_C348V	forward	5'-CCATGCTGAACTTCGTCCAGATGGGATCG-3'
	reverse	5'-CGATCCCATCTGGACGAAGTTCAGCATGG-3'
PylRS_C348W	forward	5'-ATGCTGAACTTCTGGCAGATGGGATCG-3'
	reverse	5'-CGATCCCATCTGCCAGAAGTTCAGCAT-3'
PylRS_C348Y	forward	5'-CCATGCTGAACTTCTACCAGATGGGATCG-3'
	reverse	5'-CGATCCCATCTGGTAGAAGTTCAGCATGG-3'
PylRS_Y384F	forward	5'-CCTGCATGGTCTTTGGGGATACCC-3'
	reverse	5'-GGGTATCCCCAAAGACCATGCAGG-3'
PylRS_L309A	forward	5'-CAAACCTTTACAACTACGCGCGCAAGCTTG-3'
	reverse	5'-CAAGCTTGCGCGCGTAGTTGTAAAGGTTTG-3'
PylRS_Y306A	forward	5'-GCTCCAAACCTTGCCAACTACCTGCG-3'
	reverse	5'-CGCAGGTAGTTGGCAAGGTTTGGAGC-3'
PylRS_Y306A_L309A	forward	5'-ACCTTGCCAACTACGCGCGCAAGCTTG-3'
	reverse	5'-CAAGCTTGCGCGCGTAGTTGGCAAGGT-3'

Synthesis of ester type photo-cross-linkable amino acids

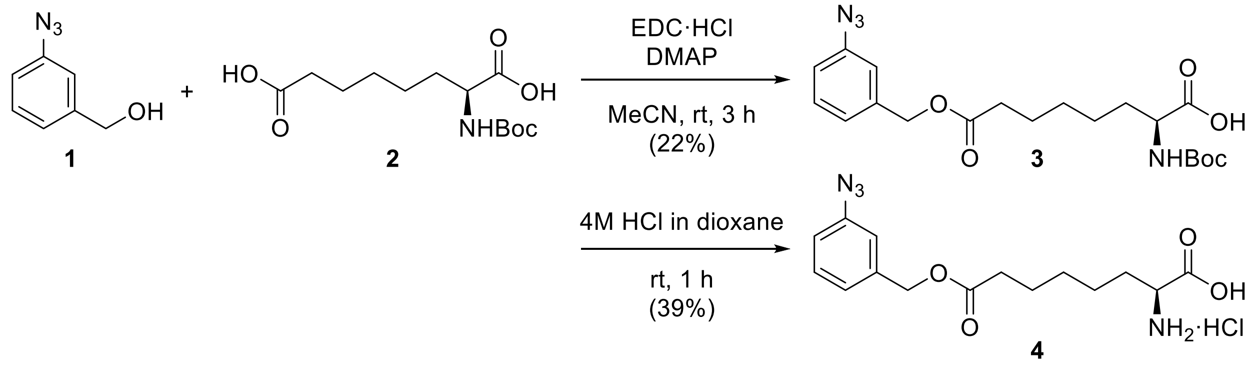

Scheme S1. Synthesis of AzAsu (**4**).

Synthesis of compound **3**

Compound **2** (2.50 g, 8.64 mmol), EDC·HCl (2.15 g, 11.2 mmol), and DMAP (106 mg, 0.87 mmol) were added to a solution of compound **1** (1.29 g, 8.65 mmol) in anhydrous MeCN (32 mL) at room temperature, and the mixture was stirred at room temperature for 3 h under Ar atmosphere. The R_f value of compound **3** was around 0.1 to 0.2 in thin-layer chromatography with hexane-AcOEt-AcOH (500/200/1) as eluent. The resultant mixture was quenched by an addition of water, and extracted with AcOEt. The combined organic layer was washed with brine, and dried over Na_2SO_4, and the solvent was removed under reduced pressure. The residue was purified several times by silica-gel column chromatography (hexane/AcOEt/AcOH = 500/200/1) to afford **3** (815 mg, 1.94 mmol, 22%) as a colorless oil. **3**: ^{1}H-NMR ($CDCl_3$) δ 1.37 (m, 4H), 1.44 (s, 9H) , 1.66 (m, 3H) , 1.83 (m, 1H), 2.37 (t, *J* = 7.3 Hz, 2H), 4.11 (m, 0.3H*), 4.29 (m, 0.7H*), 5.06 (br, 0.7H*), 5.09 (s, 2H), 6.28 (br, 0.3H*), 6.99 (d, *J* = 7.8 Hz, 1H), 7.00 (s, 1H), 7.11 (d, *J* = 7.8 Hz, 1H), 7.34 (t, *J* = 7.8 Hz, 2H), 8.34 (br, 1H); ^{13}C-NMR (CD_3Cl) δ: 14.3, 21.2, 24.7, 25.1, 28.4, 28.8, 32.4, 33.5, 34.2, 60.6, 65.6, 80.3, 118.6, 118.8, 124.6, 130.1, 138.2, 140.5, 155.8, 166.2, 171.5, 173.5; HRMS (FAB) Calcd. for $C_{20}H_{29}N_4O_6$ (MH^+): 421.2087, Found 421.2082.

*Atropisomers based on the amide bond were observed in a ratio of 7:3.

Synthesis of compound **4**

A solution of HCl in dioxane (4 mL) was added to compound **3** (280 mg, 0.67 mmol) at room temperature, and the resulting mixture was stirred at room temperature for 1 h. The R_f value of compound **4** was around 0.3 in thin-layer chromatography with $CHCl_3$-MeOH (10/1) as eluent. The solvent was removed under reduced pressure, and the residue was purified by silica-gel chromatography ($CHCl_3$/MeOH = 20/1) to afford **4** (92 mg, 0.26 mmol, 39%) as a white solid. **4**: ^{1}H-NMR (CD_3OD) δ: 1.28-1.49 (m, 4H), 1.67 (m, 2H), 1.87-1.93 (m, 2H), 2.41 (t, J = 7.3 Hz, 2H), 3.94 (m, 1H), 5.11 (s, 2H), 7.03 (d, J = 7.8 Hz, 1H), 7.04 (s, 1H), 7.16 (d, J = 7.8 Hz, 1H), 7.38 (t, J = 7.8 Hz, 2H); ^{13}C-NMR (CD_3OD) δ: 25.4, 25.4, 29.3, 31.1, 34.5, 49.0, 66.2, 119.3, 119.4, 125.4, 130.9, 139.7, 141.5, 174.7; HRMS (MALDI-TOF) Calcd. for $C_{15}H_{21}N_4O_4$ (MH-HCl$^+$): 321.1563, Found 321.1557.

^{1}H NMR (400 MHz) of compound **3** in $CDCl_3$

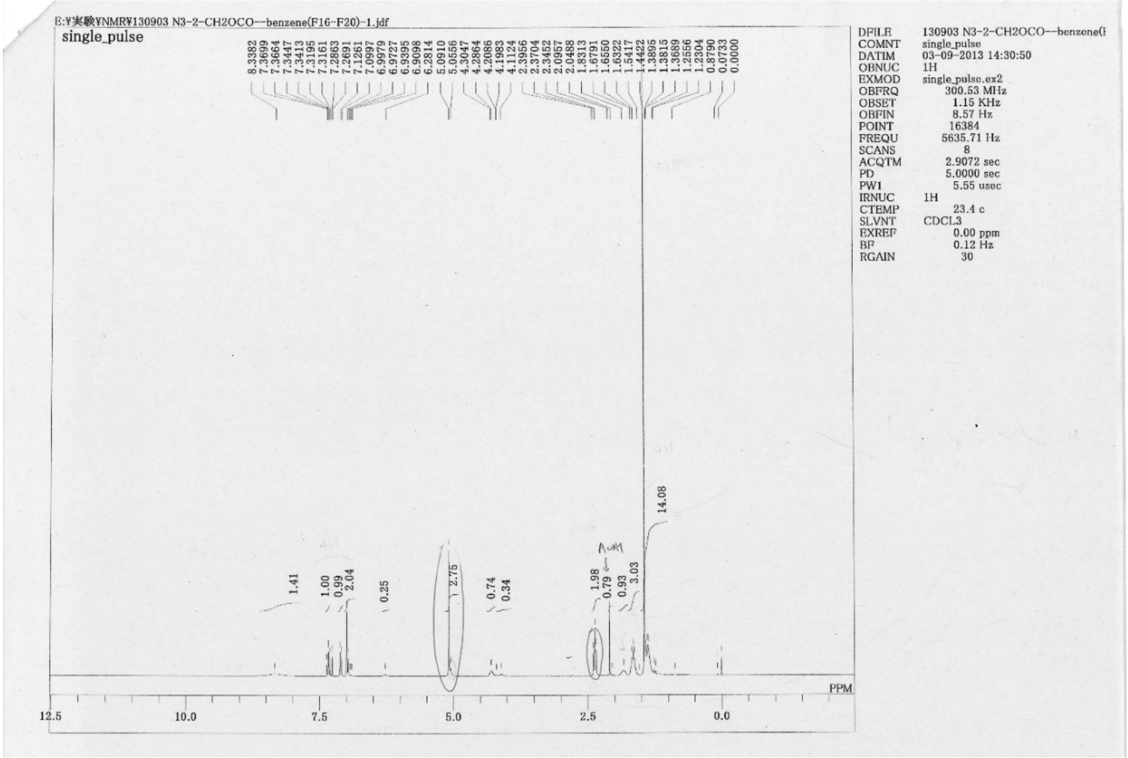

^{13}C NMR (101 MHz) of compound **3** in $CDCl_3$

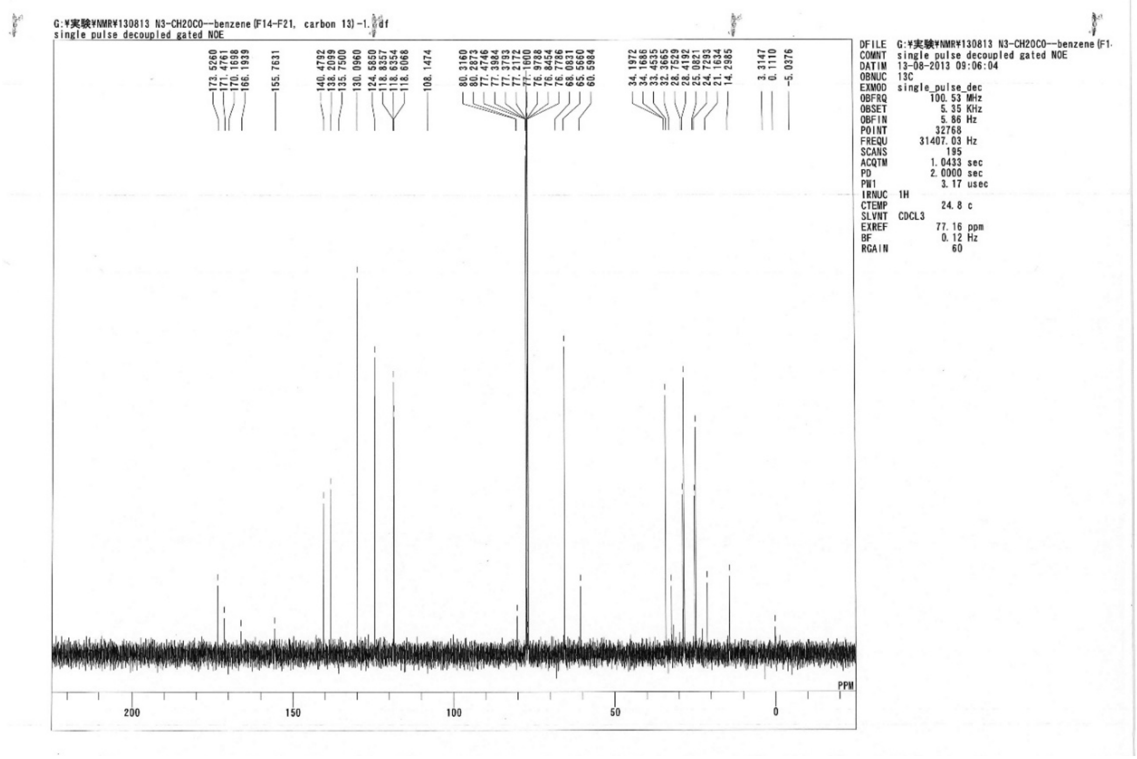

^{1}H NMR (400 MHz) of compound **4** in CD_3OD

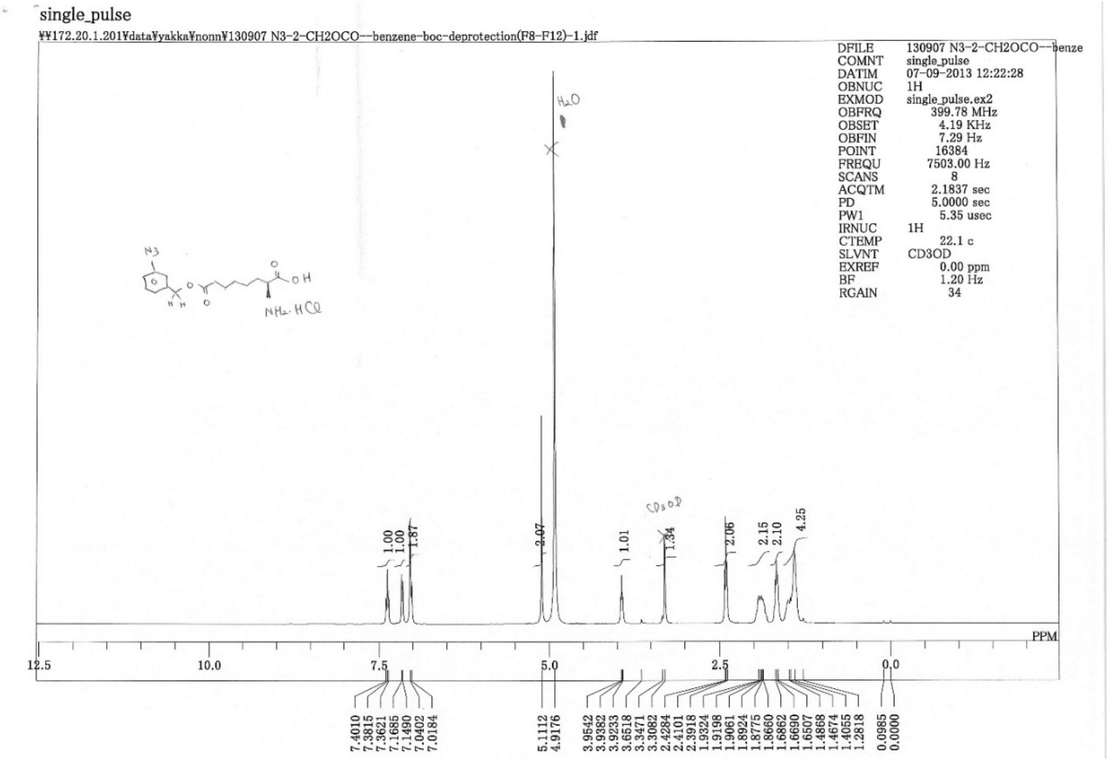

^{13}C NMR (101 MHz) of compound **4** in CD_3OD

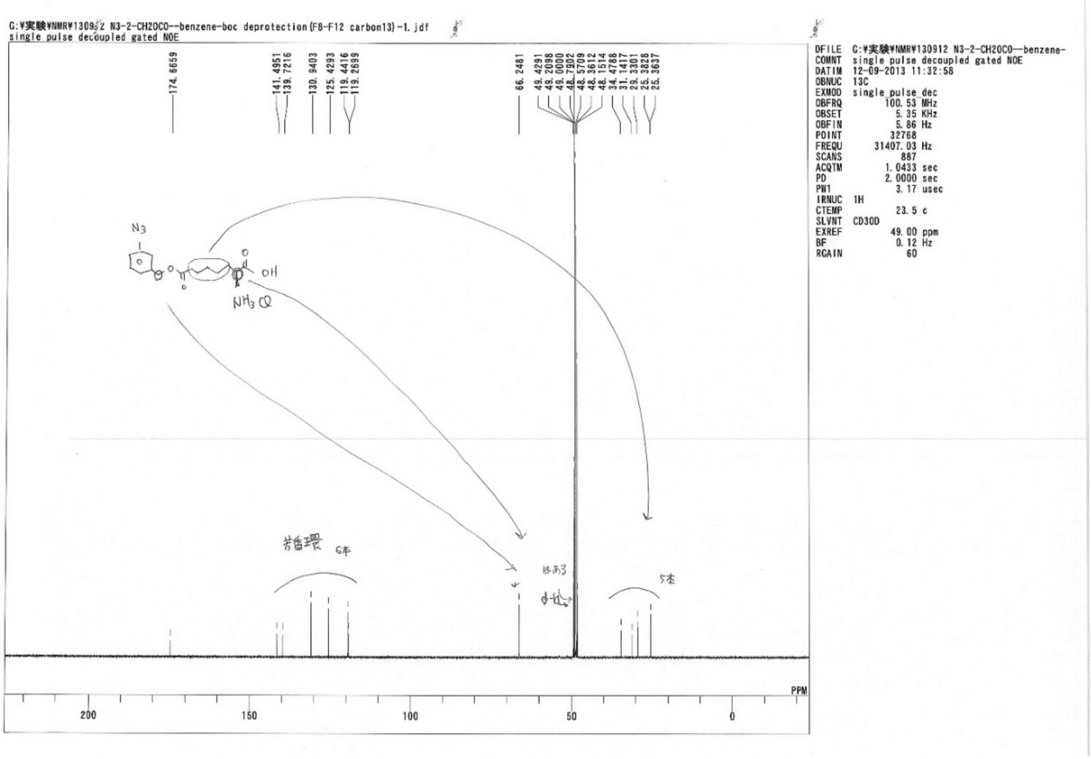

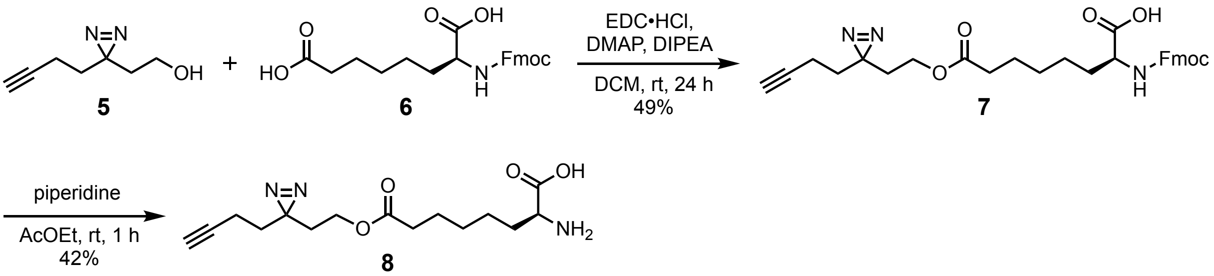

Scheme S2. Synthesis of DiZAAsu (**8**).

Synthesis of compound **7**

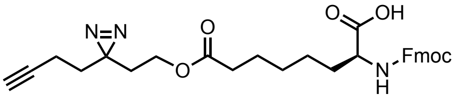

2-(3-But-3-ynyl-3*H*-diazirin-3-yl)ethanol (**5**) (50 mg, 0.36 mmol), DIPEA (189 μL, 1.2 mmol), EDC·HCl (104 mg, 0.54 mmol), and DMAP (4.4 mg, 0.036 mmol) were added to a solution of (*S*)-2-((((9*H*-fluoren-9-yl)methoxy)carbonyl)amino)octanedioic acid (**6**) (224 mg, 0.54 mmol) in anhydrous DCM (7.5 mL) at 0 °C, and the mixture was stirred at room temperature for 24 h under an Ar atmosphere. The reaction mixture was added water and extracted with AcOEt. The combined organic layers were dried over Na_2SO_4, and the solvent was removed under reduced pressure. The residue was purified by silica-gel column chromatography ($CHCl_3$/MeOH = 40:1) to afford **7** (94 mg, 0.18 mmol, 49%) as a white solid. Notably, NMR analysis revealed that this compound exists as two rotamers in an 8:2 ratio.

7: ^{1}H NMR (400 MHz, $CDCl_3$) δ 1.20–1.50 (m, 4.4H), 1.53–1.78 (m, 6.9H), 1.81–2.05 (m, 3.7H), 2.31 (t, *J* = 9.0 Hz, 2H), 3.95 (t, *J* = 8.8 Hz, 2H), 4.00–4.11 (m, 0.2H), 4.21 (t, *J* = 9.2 Hz, 1H), 4.30–4.55 (m, 2.8H), 5.48 (d, *J* = 10.8 Hz, 0.8H), 6.36 (d, *J* = 10.8 Hz, 0.2H), 7.29 (t, *J* = 10.0 Hz, 2H), 7.38 (t, *J* = 10.0 Hz, 2H), 7.49–7.62 (m, 2H), 7.74 (d, *J* = 9.8 Hz, 2H), 8.39 (br, 1H); ^{13}C NMR (76 MHz, CD_3Cl) δ 13.4, 24.7, 25.2, 26.5, 28.9, 32.4, 34.2, 47.3, 53.7, 54.7, 59.1, 67.1, 67.6, 69.6, 82.7, 120.1, 125.3, 127.2, 127.8, 141.4, 144.0, 144.1, 156.5, 173.7, 177.1; HRMS (MALDI-TOF) Calcd. for $C_{30}H_{33}N_3O_6Na$: 554.2267, Found 554.2263.

Synthesis of compound **8**

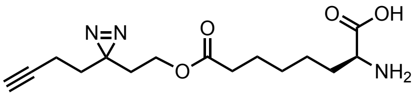

Piperidine (107 µL, 1.1 mmol) was added to a solution of **7** (94 mg, 0.18 mmol) in AcOEt (1.0 mL), and the resulting mixture was stirred at room temperature for 1 h under an Ar atmosphere. The solvent was removed under reduced pressure, and the residue was purified by silica-gel chromatography ($CHCl_3$/MeOH = 4:1) to afford **8** (23 mg, 0.08 mmol, 42%) as a pale-yellow solid. **8**: ^{1}H NMR (300 MHz, CD_3OD) δ 1.35–1.52 (m, 4H), 1.59–1.74 (m, 2H), 1.64 (t, *J* = 7.5 Hz, 2H), 1.75 (t, *J* = 6.1 Hz, 2H), 1.75–1.95 (m, 2H), 2.03 (dt, *J* = 2.7, 7.5 Hz, 2H), 2.29 (t, *J* = 2.7 Hz, 1H), 2.36 (t, *J* = 7.5 Hz, 2H), 3.52 (dd, *J* = 5.1, 7.1 Hz, 1H), 3.97 (t, *J* = 6.3 Hz, 2H); ^{13}C NMR (76 MHz, CD_3OD) δ 13.8, 25.7, 26.0, 27.5, 29.9, 32.3, 33.1, 33.5, 34.9, 56.2, 60.3, 70.4, 83.6, 174.6, 175.0; HRMS (MALDI-TOF) Calcd. for $C_{15}H_{24}N_3O_4$: 310.1767, Found 310.1761.

^{1}H NMR (400 MHz) of compound **7** in $CDCl_3$

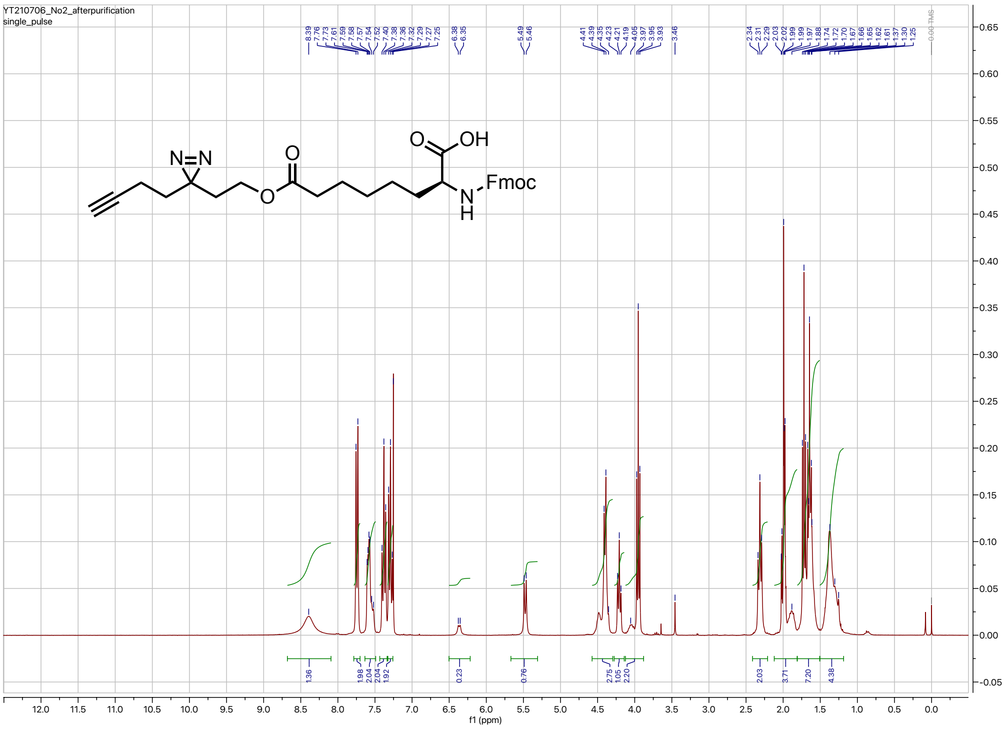

^{13}C NMR (76 MHz) of compound **7** in CD_3Cl

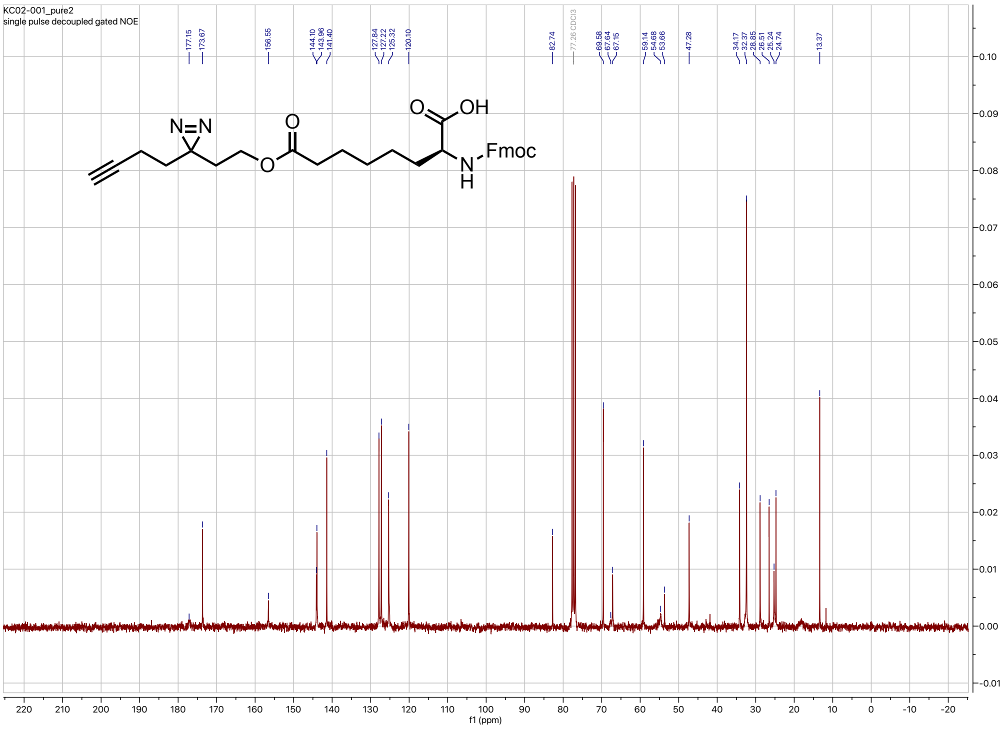

^{1}H NMR (300 MHz) of compound **8** in CD_3OD

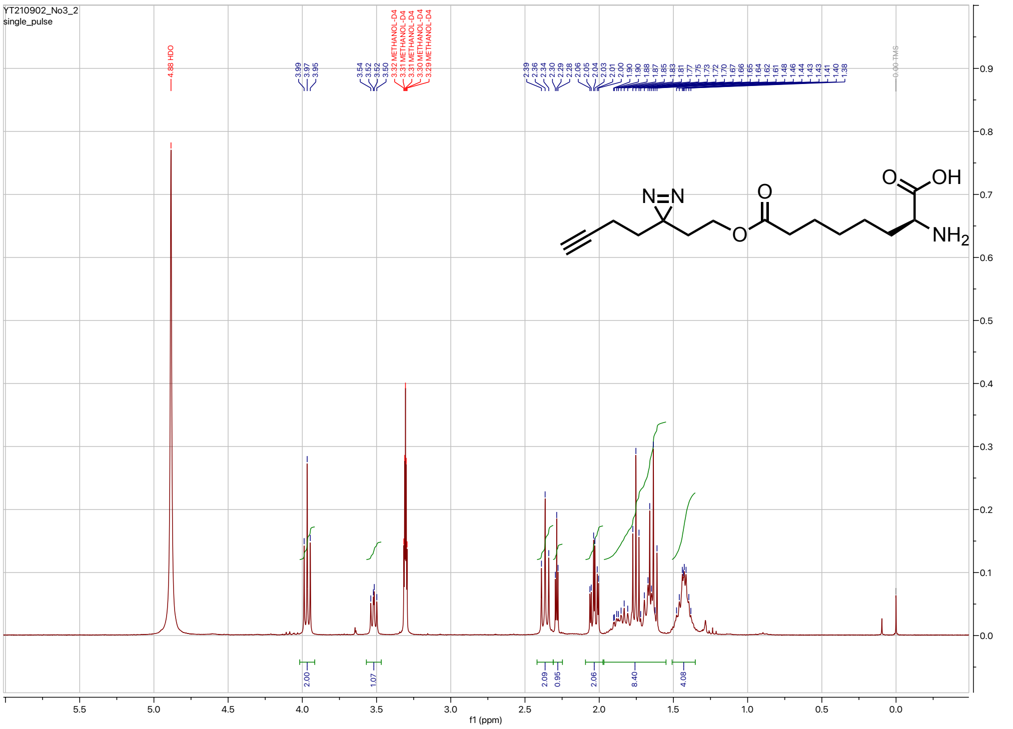

^{13}C NMR (76 MHz) of compound **8** in CD_3OD

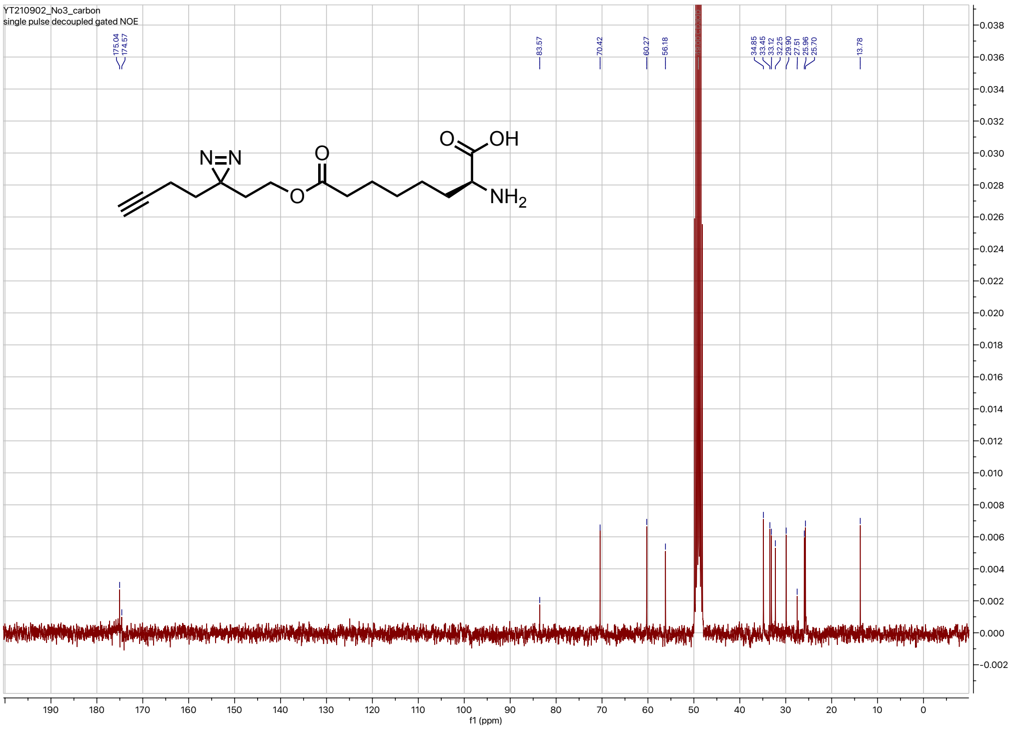

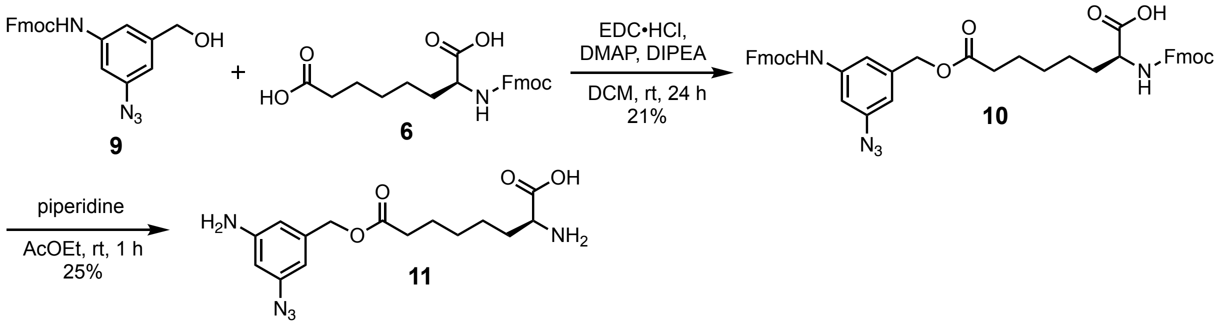

Scheme S3. Synthesis of mAAAsu (**11)**.

Synthesis of compound **10**

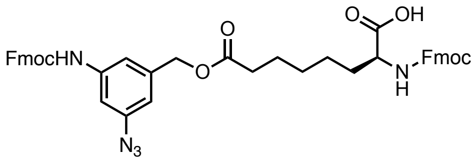

(9*H*-Fluoren-9-yl)methyl (3-azido-5-(hydroxymethyl)phenyl)carbamate (**9**) [3] (0.49 g, 1.3 mmol), DIPEA (0.66 mL, 3.8 mmol), EDC·HCl (0.36 g, 1.9 mmol), and DMAP (16 mg, 0.13 mmol) were added to a solution of (*S*)-2-((((9*H*-fluoren-9-yl)methoxy)carbonyl)amino)octanedioic acid (**6**) (0.74 g, 1.8 mmol) in anhydrous DCM (7.5 mL) at 0 °C, and the mixture was stirred at room temperature for 24 h under an Ar atmosphere. The reaction mixture was then concentrated under reduced pressure. The resulting residue was purified by silica-gel column chromatography ($CHCl_3$/MeOH = 50:1) to afford **10** (0.21 g, 0.27 mmol, 21%) as a white solid.

10: ^{1}H NMR (300 MHz, $CDCl_3$) δ 1.23–1.50 (m, 4H), 1.50–1.78 (m, 4H), 2.34 (t, *J* = 7.2 Hz, 2H), 4.13–4.27 (m, 2H), 4.38 (d, *J* = 7.2 Hz, 2H), 4.51 (d, *J* = 6.6 Hz, 2H), 4.95–5.10 (m, 2H), 5.40 (d, *J* = 8.1 Hz, 1H), 6.66 (s, 1H), 7.03 (brs, 1H), 7.13 (brs, 1H), 7.21–7.43 (m, 9H), 7.48–7.62 (m, 4H), 7.73 (dd, *J* = 7.5 Hz, 4H).

Synthesis of compound **11**

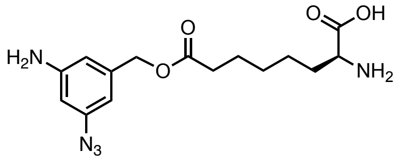

Piperidine (78 µL, 0.79 mmol) was added to a solution of **10** (206 mg, 0.26 mmol) in AcOEt (0.31 mL), and the resulting mixture was stirred at room temperature for 1 h under an Ar atmosphere. The solvent was removed under reduced pressure, and the residue was washed with AcOEt and $CHCl_3$ to afford **11** (22 mg, 66 µmol, 25%) as a pale-yellow solid.

11: ^1H NMR (300 MHz, CD_3OD) δ 1.32–1.52 (m, 4H), 1.58–1.93 (m, 4H), 2.39 (t, *J* = 7.2 Hz, 2H), 3.48–3.57 (m, 1H), 4.98 (s, 2H), 6.28–6.36 (m, 2H), 6.28–6.36 (dd, *J* = 1.7 Hz, 1H).

^{1}H NMR (300 MHz) of compound **10** in $CDCl_3$

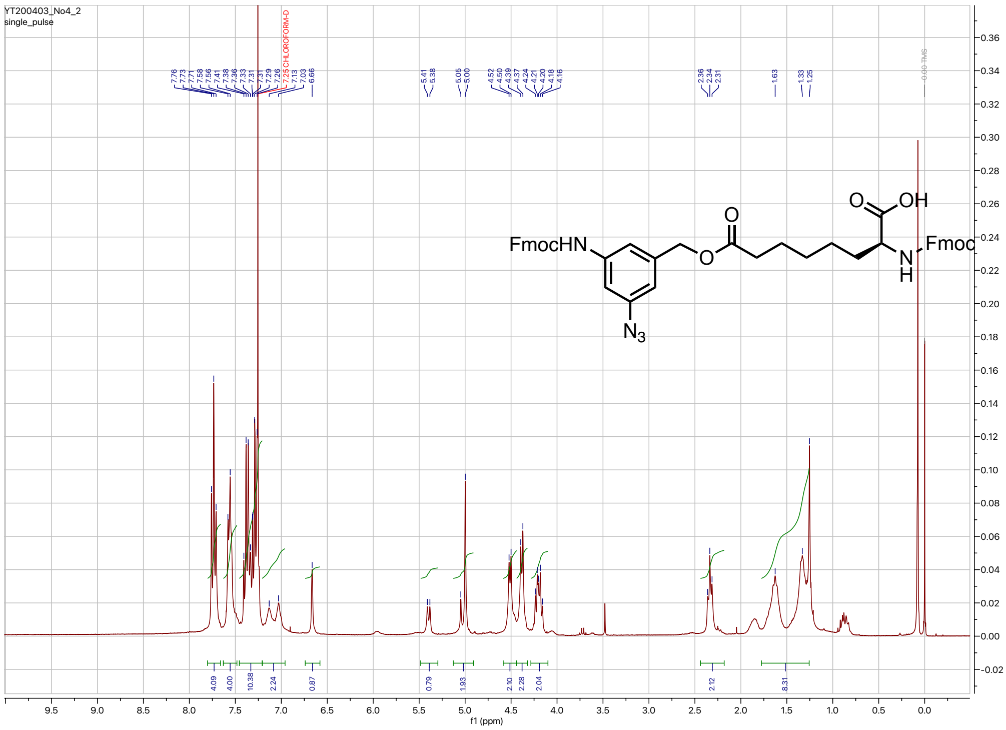

^{1}H NMR (300 MHz) of compound **11** in CD_3OD

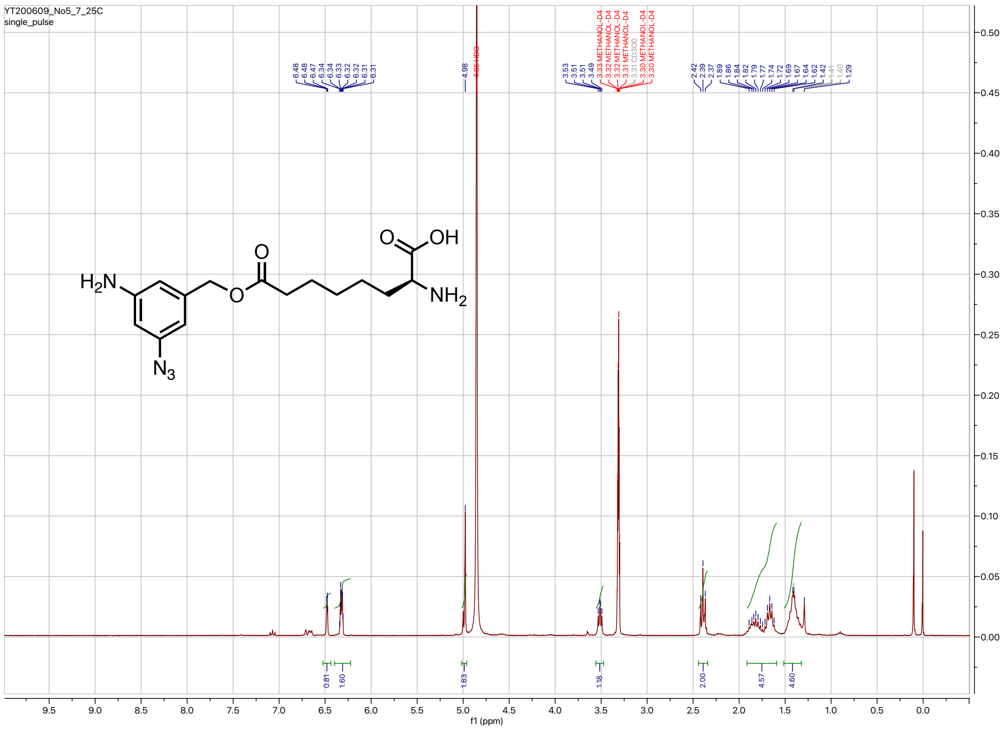

References

[1] S. Moggridge, P. H. Sorensen, G. B. Morin, C. S. Hughes, ”Extending the Compatibility of the SP3 Paramagnetic Bead Processing Approach for Proteomics” *J. Proteome Res.* 2018, **17**, 1730.

[2] T. S. Batth, M. X. Tollenaere, P. Ruther, A. Gonzalez-Franquesa, B. S. Prabhakar, S. Bekker-Jensen, A. S. Deshmukh, J. V. Olsen, ”Protein Aggregation Capture on Microparticles Enables Multipurpose Proteomics Sample Preparation” *Mol. Cell. Proteomics* 2019, **18**, 1027.

[3] A. Yamaguchi, T. Matsuda, K. Ohtake, T. Yanagisawa, S. Yokoyama, Y. Fujiwara, T. Watanabe, T. Hohsaka, K. Sakamoto, ”Incorporation of a Doubly Functionalized Synthetic Amino Acid into Proteins for Creating Chemical and Light-Induced Conjugates” *Bioconjug. Chem.* 2016, **27**, 198.
